# Synthesis of spiroimidazopyridineoxindole, spiropyridopyrimidineoxindole and spiropyridodiazepineoxindole derivatives based on heterocyclic ketene aminals *via* a four-component reaction†

**DOI:** 10.1039/c8ra10379h

**Published:** 2019-05-24

**Authors:** Fatemeh Rahimi, Mohammad Bayat, Hajar Hosseini

**Affiliations:** Department of Chemistry, Faculty of Science, Imam Khomeini International University Qazvin Iran bayat_mo@yahoo.com m.bayat@sci.ikiu.ac.ir +98 28 33780040

## Abstract

Here, we have described the synthesis of novel spiropyridineoxindole derivatives containing a pyridone ring *via* a four-component reaction between various diamines, 1,1-bis(methylthio)-2-nitroethylene, isatin derivatives and Meldrum's acid in the presence of *p*-toluenesulfonic acid. This protocol has some advantages such as the availability of starting materials, good yields, facile separation of products, the use of ethanol as an environmentally benign solvent and easy formation of three new bonds in one operation.

## Abstract 2

A simple and efficient approach to the synthesis of three new classes of fused spiropyridineoxindoles using heterocyclic ketene aminals.

## Graphical abstract

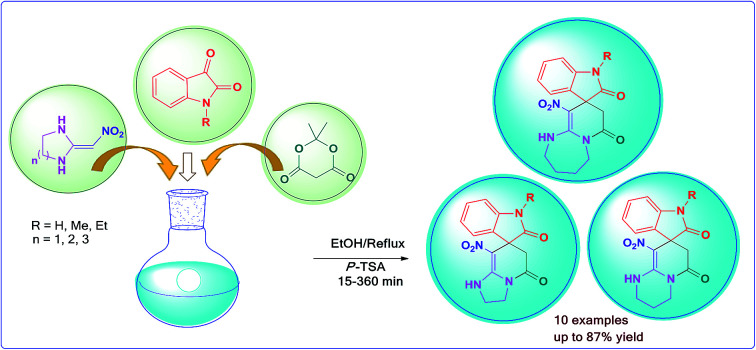

## Introduction

The indole moiety is the most well-known heterocycle and a common and important feature of a variety of natural products and medicinal agents.^1^ Furthermore, it has been reported that the addition of the indole 3-carbon atom in the formation of spiroindoline derivatives highly enhances biological activity.^2^

Spirooxindoles are a key structural element in a wide range of natural products with biological activities.^3^ They have attracted significant attention due to their useful pharmacological properties and biological activities including anti-microbial,^4^ anti-tumor,^5^ anti-tubercular,^6^ anti-inflammatory,^7^ anti-HIV,^8^ anti-fungal,^9^ the action as inhibitors of the human NK-1 receptor,^10^ anti-cancer,^11^ antibiotic,^12^ and anti-malarial.^13^

**Fig. 1:**
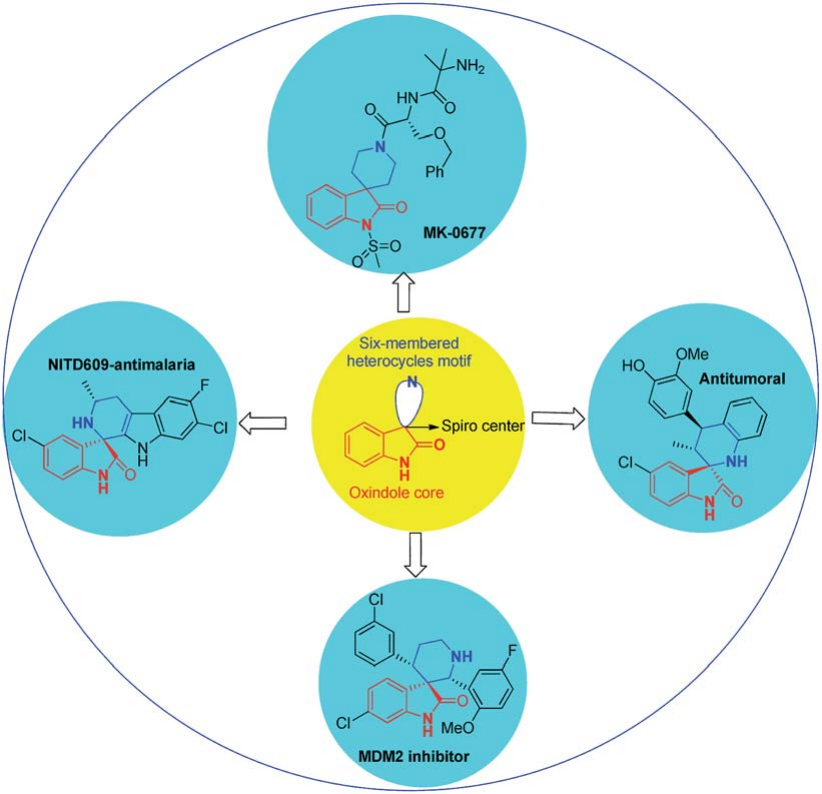
Bioactive and medicinally important compounds containing a spiropyridineoxindole skeleton.

Spirocyclic oxindoles containing a six-membered moiety, especially a six-membered piperidine structure at the C-3 position, have a wide spectrum of biological activities; examples include the non-peptidyl growth hormone secretagogue MK-0677 (ref. 14) and potent non-peptide MDM2 inhibitors,^15^ which may have utility as anticancer agents (Fig. 1).

During the last years, there has been considerable interest in the synthesis of spirooxindoles fused in the 3-position from three-membered to seven-membered spiro-rings^16^ and other fused heterocyclic compounds, which have multi-ring structures similar to spirooxindole dihydropyridines and spirooxindolepyrans.^17^

**Fig. 2:**
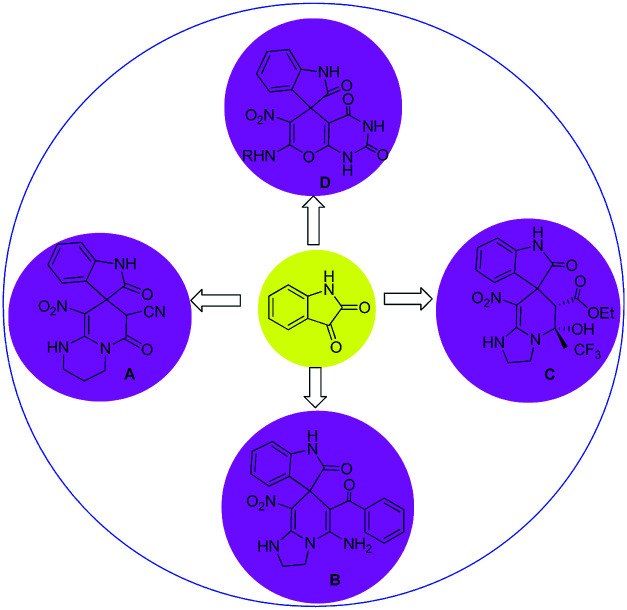
Summary of previous works of spiropyridineoxindole synthesis.

In general, the synthesis of spirooxindole frameworks containing six-membered nitrogen rings has more limitations than that of five-membered heterocyclic moieties. In the last decade, synthetic methods for the generation of spiropyridineoxindoles *via* multicomponent reactions have been abundantly developed.^18^ Previous approaches for producing these structures relied on utilizing isatin, various C–H acids and a wide variety of different enamines as starting materials. Among these strategies, similar cases in which ketene aminals have been used as enamines are reported here (Fig. 2).

In 2015, a green approach to the synthesis of the spirooxoindole derivative A was described in water in the presence of a catalytic amount of NaCl by using ethylcyanoacetate and heterocyclic ketene aminals.^19^ In 2014, another reaction was reported using benzoylacetonitrile and 2-(nitromethylene)imidazoline in ethanol and trimethylamine, which led to the corresponding product B.^20^

In 2013, a method was developed using a mixture of ethyl trifluoroacetate, HKA and different isatins in the presence of piperidine in ethanol to give product C.^21^ In 2019, the four-component reaction of various amines and nitroketene dithioacetal with isatin and barbituric acid derivatives in water afforded spirooxindole D.^22^

As a part of our program on the study of developing new multi-component reactions for the synthesis of heterocyclic compounds, we report the efficient synthesis of novel spiroimidazopyridineoxindole, spiropyridopyrimidineoxindole and spiropyridodiazepineoxindole structures *via* a one-pot, four-component reaction of nitro ketene aminals derived from the addition of various 1,*n*-diamines to 1,1-bis(methylthio)-2-nitroethylene, isatin and its derivatives and Meldrum's acid in the presence of *p*-TSA. To the best of our knowledge, these structures have not been synthesized so far and there are no reports on the preparation of spirooxindoles from Meldrum's acid.

## Results and discussion

**Scheme 1:**
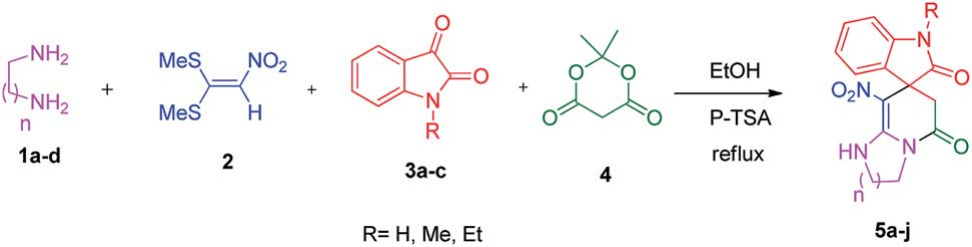
Synthetic scheme for the generation of products 5a–j.

We prepared the spiropyridineoxindole derivative 5*via* one-pot four-component condensation of diamine 1, 1,1-bis(methylthio)-2-nitroethylene 2, isatin 3 and Meldrum's acid 4 in the presence of *p*-TSA catalyst in ethanol under reflux conditions (Scheme 1). The reaction was completed after 0.15–6 h to afford the corresponding heterocyclic systems 5a–j in moderate to good yields (69–87%).

For our initial investigation, the reaction of diamine 1a (1 mmol), dithioacetal 2 (1 mmol), isatin 3a (1 mmol) and Meldrum's acid 4 (20% mmol) was considered as the model reaction. The effects of various catalysts, solvents and temperatures were monitored (Table 1). These results indicated that the best reaction conditions for the synthesis of spirooxindole 5a were obtained in ethanol as the solvent and by using *p*-TSA as the catalyst at reflux conditions. This procedure provided the highest yield of 87% and the shortest reaction time (Table 1, entry 7).

**Table 1:** Optimization of reaction conditions for the synthesis of 5a^a^

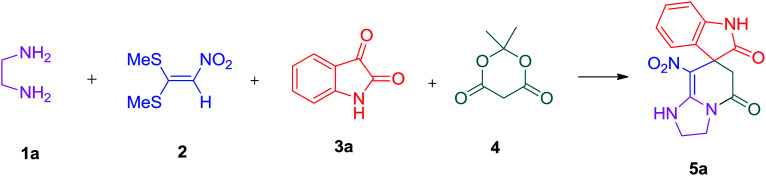					
Entry	Solvent	Catalyst (mol%)	Time (h)	Temp. (°C)	Yield^b^ (%)
1	EtOH	NEt_3_	1	80	75
2	EtOH	Piperidine	24	80	None
3	EtOH	—	5	r.t	60
4	H_2_O/EtOH (1 : 1, v/v)	—	6	80	65
5	H_2_O/EtOH (3 : 1, v/v)	—	7	80	40
6	EtOH	*p*-TSA	24	r.t	Trace
**7**	**EtOH**	***p*-TSA**	**0.15**	**80**	**87**

a The reaction was performed using 1a, 2, 3a, 4 (1 mmol), catalyst (0.2 mmol), and solvent (10 mL).

b Isolated yield based on 5a.

We explored the scope of this reaction by varying the structures of diamine 1a–d and isatin 3a–c components. The reaction proceeded to afford a series of spiropyridineoxindole derivatives 5a–j in 66–87% yields. The results are summarized in Table 2. This reaction was performed with other derivatives of isatin (*N*-benzyl and *N*-butyl isatin) under the same conditions but did not result in the product. Additionally, the reaction of *N*-ethyl isatin with 1,2-diaminopropane and 1,3-diaminopropane did not produce a product. Also, when 1,2-diaminocyclohexane was used for the synthesis of ketene aminal, no reaction occurred. We also tried using 1,2-diaminophenyl and aromatic amines in the reaction conditions. All the reactions were very slow and did not result in the desired products. The structures of compounds 5a–j were deduced from their mass, IR, ^1^H NMR, and ^13^C NMR spectroscopic data (ESI†).

**Table 2:** Compounds 5a–j^a^

Entry	Diamine	R	Product	Time (h)	Yield^b^ (%)
1	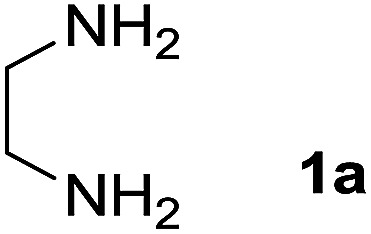	H	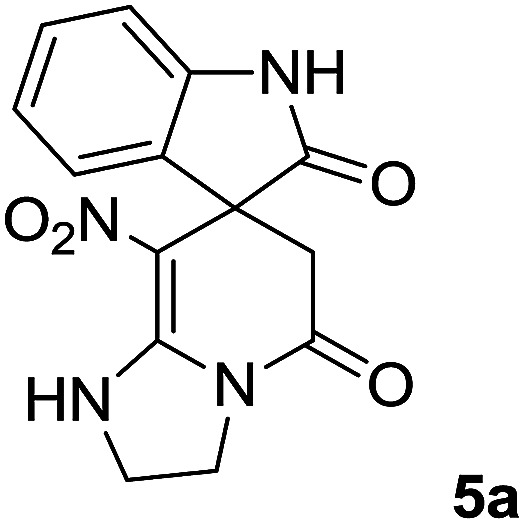	0.15	87
2	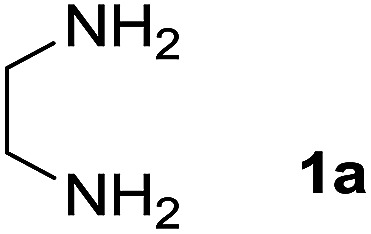	Me	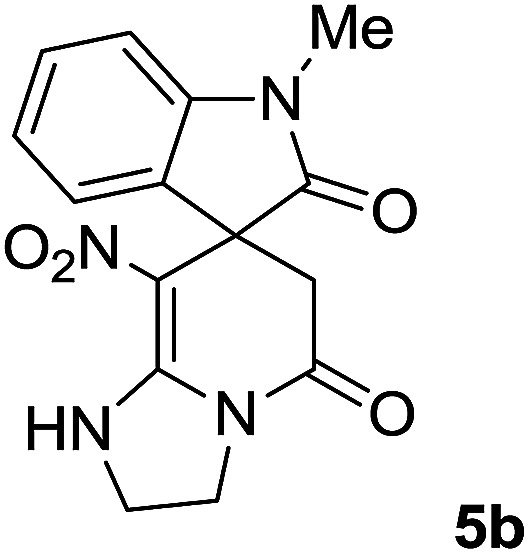	2	85
3	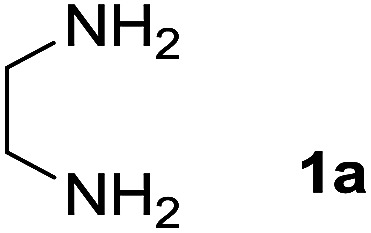	Et	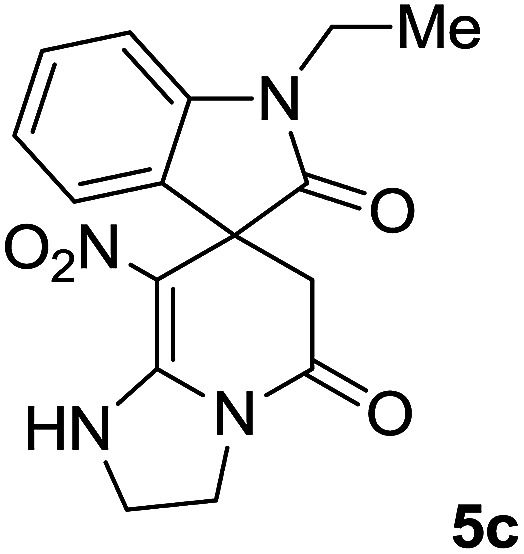	0.5	70
4	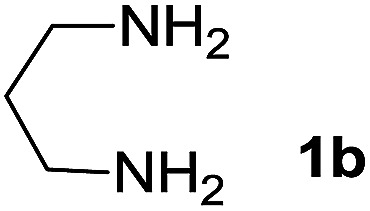	Me	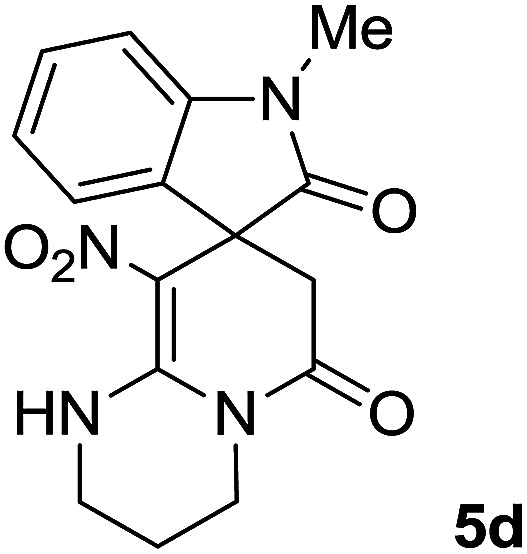	3	73
5	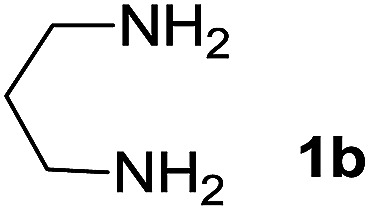	H	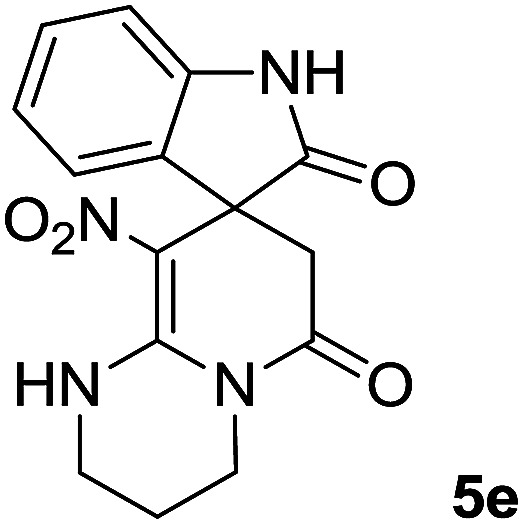	3	83
6	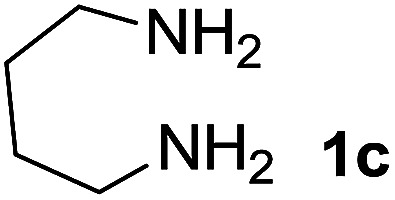	Me	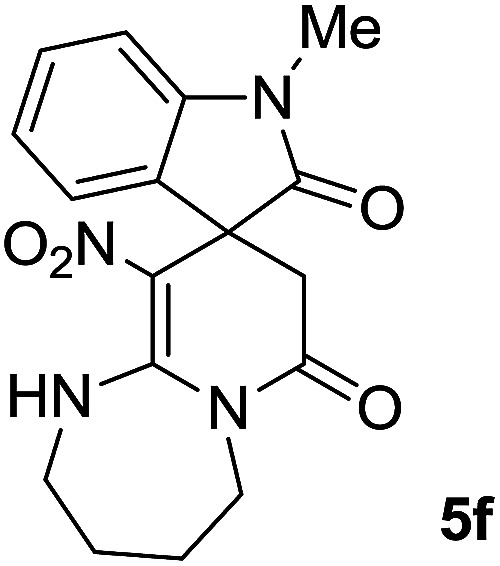	5	75
7	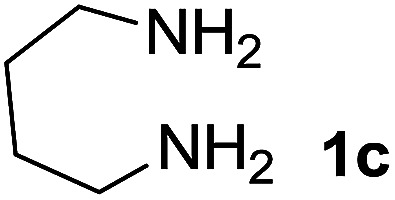	Et	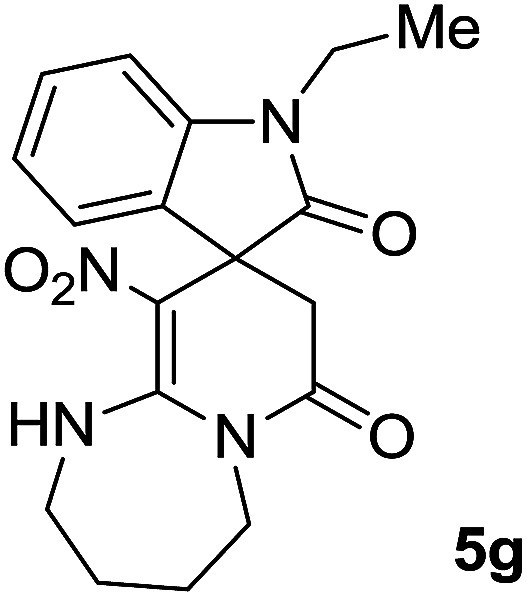	6	69
8	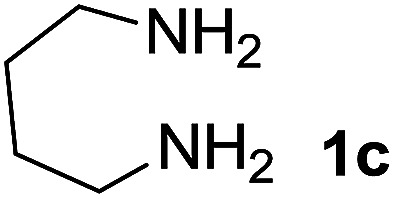	H	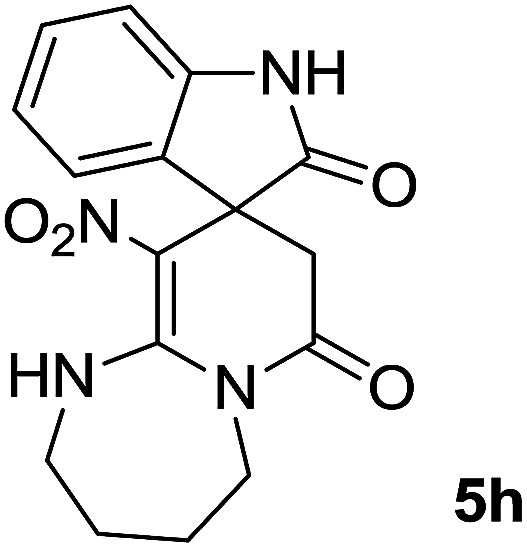	5	72
9	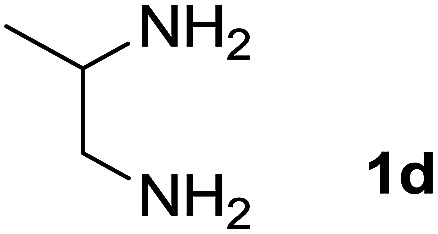	H	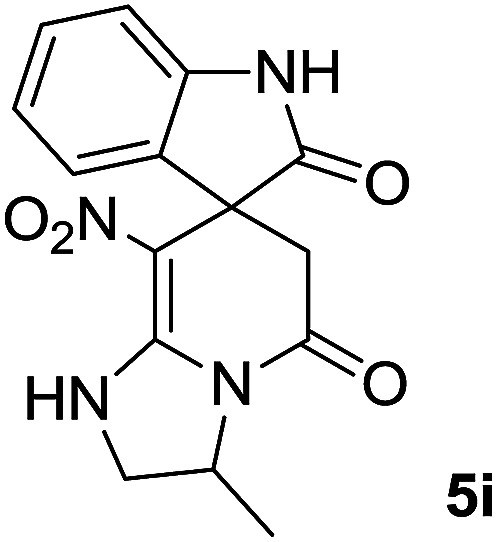	4	80
10	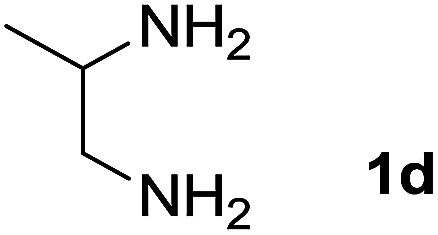	Me	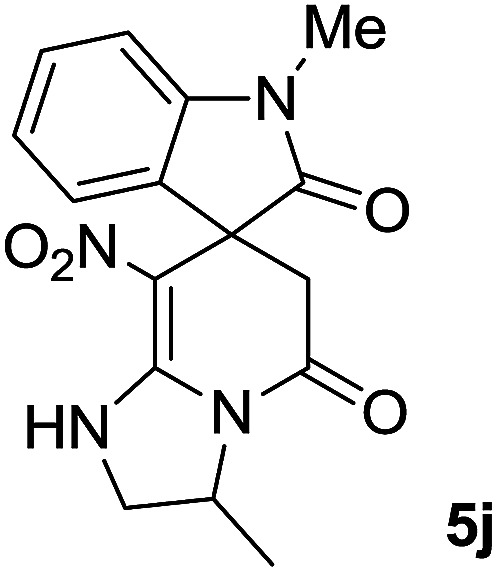	3	76

a The reaction conditions include 1, 2, 3, 4 (1 mmol), catalyst (0.2 mmol), and ethanol (10 mL).

b Yields refer to the isolated pure products.

The ^1^H and ^13^C NMR spectra of the crude products clearly indicate the formation of the suggested products 5a–j. As a representative case, the key signals of the ^1^H and ^13^C NMR chemical shifts for 8-nitro-2,3-dihydro-1*H*-spiro[imidazo[1,2-*a*]pyridine-7,3′-indoline]-2′,5(6*H*)-dione 5a are shown in the ESI.†

The ^1^H NMR spectrum of 5a shows the two NH groups (*δ* 9.57, 10.55 ppm) that are exchangeable with D_2_O. The two protons of methylene appear at *δ* 2.36 and 3.04 ppm as two doublets. Four protons of the aromatic ring can be observed at *δ* 6.81–7.20 ppm as four doublets. The ^1^H-decoupled ^13^C NMR spectrum of 5a indicates 14 distinct resonances in accordance with the desired structure. The characteristic signal of the carbon of the CH_2_ group is seen at *δ* 41.8 ppm. The carbon of the spiro center appears at *δ* 49.5 ppm. The two signals at *δ* 105.6 and 153.6 ppm are related to C–NO_2_ and C=C–NH, respectively. The two carbonyl groups appear at *δ* 165.6 and 177.6 ppm.

The mass spectrum of 5a displays a molecular-ion peak at *m*/*z* 300, which is in agreement with the proposed structure. The IR spectrum of 5a shows broad absorption bands due to NH at 3327^−1^, stretching vibrations of the CH_2_ groups at 2920^−1^, strong absorption bands of carbonyl groups at 1725^−1^ and 1683^−1^ and absorption bands at 1480^−1^ and 1369 cm^−1^ related to NO_2_.

**Scheme 2:**
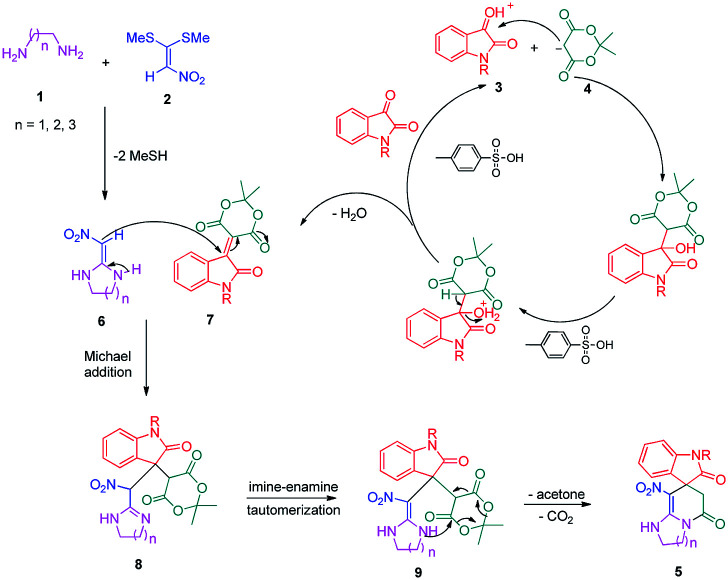
Proposed mechanism for the formation of the product 5a.

A plausible mechanism for the formation of spiropyridineoxindole 5 is depicted in Scheme 2. On the basis of the well-established chemistry of 1,1-bis(methylthio)-2-nitroethylene, initially, the addition of diamine 1 to 1,1-bis(methylthio)-2-nitroethylene 2 leads to the formation of ketene aminal 6. The second step involves the condensation of isatin 3 with Meldrum's acid 4 in the presence of *p*-toluenesulfonic acid to afford the Knoevenagel product 7. Then, the Michael addition of the ketene aminal 6 to adduct 7 affords the intermediate 8; it undergoes subsequent imine–enamine tautomerization, followed by intramolecular cyclization *via* the nucleophilic addition of –NH to the carbonyl group. Subsequently, one equivalent of CO_2_ and acetone are removed from intermediate 9 to give the corresponding product 5 (Scheme 2).

## Experimental

### Materials

Meldrum's acid, 1,1-bis(methylthio)-2-nitro ethylene, diamines, various isatin derivatives, *p*-toluenesulfonic acid and solvents were purchased from Sigma-Aldrich and used as received without further purification. Melting points (mp) were determined with an electrothermal 9100 apparatus. Infrared (IR) spectra were recorded on a Bruker Tensor 27 spectrometer. Nuclear magnetic resonance (NMR) spectra were obtained on a Bruker DRX-300 Avance instrument (300 MHz for ^1^H and 75.4 MHz for ^13^C) with CDCl_3_ and DMSO as solvents. Chemical shifts were expressed in parts per million (ppm) relative to internal TMS, and the coupling constant (*J*) was reported in hertz (Hz). All of the compounds were analyzed for mass data using Agilent 5975C VL MSD with a Triple-Axis Detector operating at an ionization potential of 70 eV.

### General procedure for the synthesis of 8-nitro-2,3-dihydro-1*H*-spiro[imidazo[1,2-*a*]pyridine-7,3′-indoline]-2′,5(6*H*)-dione (5a)

A mixture of ethylene diamine (66 mL, 1 mmol), 1,1-bis(methylthio)-2-nitroethylene (0.165 g, 1 mmol) and 10 mL EtOH in a 50 mL flask was refluxed for 6 h. After completion of the reaction (monitored by TLC, ethyl acetate/*n*-hexane, 1 : 1), isatin (0.147 g, 1 mmol), Meldrum's acid (0.144 g, 1 mmol) and *p*-toluenesulfonic acid (0.03 g, 0.2 mmol) were added to the reaction mixture, and it was stirred under reflux for 0.15 h. The progress of the reaction was monitored by TLC using ethyl acetate–*n*-hexane (1 : 1) as an eluent. After reaction completion, the precipitated product was collected by filtration and washed with EtOH to give the pure product 5a in 87% yield.

#### 8-Nitro-2,3-dihydro-1*H*-spiro[imidazo[1,2-*a*]pyridine-7,3′-indoline]-2′,5(6*H*)-dione (5a)

Light gray powder; yield: 0.26 g, (87%); mp: 310–312 °C; IR (KBr) (*ῡ*_max_/cm^−1^): 3327 (NH), 2920 (CH_2_), 1725, 1683 (2C=O), 1632 (C=C), 1480 and 1369 (NO_2_), 1277 (C–N); ^1^H NMR (300 MHz, DMSO): *δ* = 2.36 (1H, d, ^2^*J*_HH_ = 16 Hz, CH_2_CO), 3.03 (1H, d, ^2^*J*_HH_ = 16 Hz, CH_2_CO), 3.78–3.83 (2H, m, CH_2_NH), 3.90–4.06 (2H, m, CH_2_N), 6.82 (1H, d, ^3^*J*_HH_ = 7.8 Hz, ArH), 6.88 (1H, d, ^3^*J*_HH_ = 7.5 Hz, ArH), 7.11–7.20 (2H, m, ArH), 9.57 (1H, br, NHCH_2_), 10.55 (1H, s, NH–C=O); ^13^C NMR (75 MHz, DMSO-*d*_6_); *δ* = 41.8 (CH_2_CO), 43.2 (CH_2_NH), 44.3 (CH_2_N), 49.5 (C_spiro_), 105.6 (C–NO_2_), 110.1, 121.9, 122.7, 129.0 (4CH of Ar), 131.9, 142.1 (C–Ar), 153.6 (**C**=C–NO_2_), 165.6 (NH–**C**=O), 177.6 (CH_2_–**C**=O); MS (EI, 70 eV): *m*/*z* (%) = 300 (56) [M]^+^, 254 (69), 239 (100), 225 (24), 198 (27), 155 (14), 69 (3), 44 (20). Anal. calcd for C_14_H_12_N_4_O_4_ (300.27): C, 56; H, 4.03; N, 18.66. Found C, 56.3; H, 4.2; N, 18.50.

#### 1′-Methyl-8-nitro-2,3-dihydro-1*H*-spiro[imidazo[1,2-*a*]pyridine-7,3′-indoline]-2′,5(6*H*)-dione (5b)

White powder; yield: 0.27 g, (85%) mp: 328–330 °C; IR (KBr) (*ῡ*_max_/cm^−1^): 3334 (NH), 2886 (CH_3_), 1711, 1636 (2C=O), 1488 and 1326 (NO_2_), 1157 (C–N); ^1^H NMR (300 MHz, DMSO): *δ* = 2.17 (1H, d, ^2^*J*_HH_ = 20 Hz, CH_2_CO), 3.04 (1H, d, ^2^*J*_HH_ = 20 Hz, CH_2_CO), 3.21 (3H, s, CH_3_), 3.67–3.78 (2H, m, CH_2_NH), 3.90–4.07 (2H, m, CH_2_N), 6.82–6.98 (2H, m, ArH), 7.11–7.30 (2H, m, ArH), 9.8 (1H, s, NH); ^13^C NMR (75 MHz, DMSO-*d*_6_); *δ* = 26.9 (N–CH_3_), 41.5 (CH_2_CO), 43.3 (CH_2_NH), 44.3 (CH_2_N), 49.1 (C_spiro_), 105.5 (C–NO_2_), 109.0, 122.4, 122.6, 129.1 (4CH of Ar), 131.1, 143.6 (C–Ar), 153.6 (**C**=C–NO_2_), 165.5 (NMe–**C**=O), 175.9 (CH_2_–**C**=O); MS (EI, 70 eV): *m*/*z* (%) = 314 (82) [M]^+^, 269 (12.5), 253 (100), 225 (17), 169 (12), 130 (13), 44 (14). Anal. calcd for C_15_H_14_N_4_O_4_ (314.30): C, 57.32; H, 4.4; N; 17.83. Found C, 57.6; H, 4.1; N, 18.60.

#### 1′-Ethyl-8-nitro-2,3-dihydro-1*H*-spiro[imidazo[1,2-*a*]pyridine-7,3′-indoline]-2′,5(6*H*)-dione (5c)

White powder; yield: 0.23 g, (70%); mp: 321–323 °C; IR (KBr) (*ῡ*_max_/cm^−1^): 3335 (NH), 2925, 2856 (C–H), 1711, 1636 (2C=O), 1487 and 1369 (NO_2_), 1158 (C–N); ^1^H NMR (300 MHz, DMSO): *δ* = 1.11 (3H, t, ^3^*J*_HH_ = 6.9 Hz, CH_3_), 2.30 (1H, d, ^2^*J*_HH_ = 15.9 Hz, CH_2_CO), 3.05 (1H, d, ^2^*J*_HH_ = 15.9 Hz, CH_2_CO), 3.62–3.71 (2H, m, CH_2_NH), 3.77 (2H, q, ^3^*J*_HH_ = 6.9 Hz, CH_3_–CH_2_–N), 3.83–4.07 (2H, m, CH_2_N), 6.83 (1H, t, *J*_HH_ = 7.8 Hz, ArH), 6.93 (1H, d, *J*_HH_ = 7.5 Hz, ArH), 7.19 (1H, d, *J*_HH_ = 7.8 Hz, ArH), 7.27 (1H, t, *J*_HH_ = 7.5 Hz, ArH), 9.75 (1H, br, NH); ^13^C NMR (75 MHz, DMSO-*d*_6_): *δ* = 12.4 (CH_3_), 34.8 (CH_3_–CH_2_N), 41.5 (CH_2_C=O), 43.2 (CH_2_NH), 44.3 (CH_2_N), 49.0 (C_spiro_), 105.53 (C–NO_2_), 109.1, 122.4, 122.7, 129.1 (4CH of Ar), 131.2, 142.5 (C–Ar), 153.5 (C=C–NO_2_), 165.5 (N–C=O), 175.5 (CH_2_–C=O); MS (EI, 70 eV): *m*/*z* (%) = 328 (100) [M]^+^, 282 (38), 267 (72), 239 (98), 224 (22), 197 (20), 158 (15), 44 (21). Anal. calcd for C_16_H_16_N_4_O_4_ (328.32): C, 50.53; H, 4.91; N, 17.06. Found C, 50.1; H, 4.6; N, 17.30.

#### 1-Methyl-9′-nitro-1′,2′,3′,4′-tetrahydrospiro[indoline-3,8′-pyrido[1,2-*a*]pyrimidine]-2,6′(7′*H*)-dione (5d)

White powder; yield: 0.24 g, (73%); mp: 318–320 °C; IR (KBr) (*ῡ*_max_/cm^−1^): 3412 (NH), 3032 (C–H of Ar), 2921 (CH_3_), 1716, 1619 (2C=O), 1496 and 1323 (NO_2_), 1151 (C–N); ^1^H NMR (300 MHz, DMSO): *δ* = 1.95–2.09 (2H, m, CH_2_), 2.34 (1H, d, ^2^*J*_HH_ = 15.3 Hz, CH_2_CO), 3.13 (1H, d, ^2^*J*_HH_ = 15.3 Hz, CH_2_CO), 3.14 (3H, s, N–Me), 3.52–3.59 (2H, m, CH_2_NH), 3.72–3.78 (1H, m, CH_2_N), 3.88–3.95 (1H, m, CH_2_N), 6.94 (1H, t, *J*_HH_ = 7.2 Hz, ArH), 7.01 (1H, d, *J*_HH_ = 7.8 Hz, ArH), 7.09 (1H, d, *J*_HH_ = 7.2 Hz, ArH), 7.28 (1H, t, *J*_HH_ = 7.8 Hz, ArH), 11.50 (1H, s, NH); ^13^C NMR (75 MHz, CDCl_3_): *δ* = 19.0 (CH_2_), 26.9 (N–CH_3_), 39.2 (**C**H_2_–C=O), 39.5 (CH_2_NH), 40.3 (CH_2_N), 48.0 (C_spiro_), 107.8 (C–NO_2_), 108.6, 121.8, 122.6, 129.1 (4CH of Ar), 129.3, 143.4 (C–Ar), 152.7 (**C**=C–NO_2_), 166.4 (NMe–**C**=O), 175.9 (CH_2_–**C**=O). Anal. calcd for C_16_H_16_N_4_O_4_ (328.33): C, 58.53; H, 4.91; N, 17.06. Found C, 58.9; H, 4.5; N, 17.20.

#### 9′-Nitro-1′,2′,3′,4′-tetrahydrospiro[indoline-3,8′-pyrido[1,2-*a*]pyrimidine]-2,6′(7′*H*)-dione (5e)

White powder; yield: 0.26 g, (82%); mp 327–329 °C; ^1^H NMR (300 MHz, DMSO): *δ* = 1.90–2.05 (2H, m, CH_2_), 2.61 (1H, d, ^2^*J*_HH_ = 16.5 Hz, CH_2_CO), 3.29 (1H, d, ^2^*J*_HH_ = 16.5 Hz, CH_2_CO), 3.38–3.52 (4H, m, CH_2_N), 6.85–6.90 (3H, m, ArH), 7.15–7.21 (1H, m, ArH), 9.35 (1H, br, NH), 10.18 (1H, s, NH); ^13^C NMR (75 MHz, DMSO-*d*_6_): *δ* = 18.8 (CH_2_), 34.7 (CH_2_CO), 37.7 (CH_2_NH), 39.9 (CH_2_N), 49.8 (C_spiro_), 106.1 (C–NO_2_), 116.5, 120.2, 122.6, 126.1 (4CH of Ar), 129.3, 139.5 (C–Ar), 153.0 (**C**=C–NO_2_), 167.7 (N–**C**=O), 174.1 (CH_2_–**C**=O); MS (EI, 70 eV): *m*/*z* (%) = 314 (46) [M]^+^, 268 (100), 57 (41), 236 (31), 83 (30), 185 (20), 155 (19), 211 (14). Anal. calcd for C_15_H_14_N_4_O_4_ (314.30): C, 57.32; H, 4.49; N, 17.83. Found C, 57.5; H, 4.1; N, 17.50.

#### 1-Methyl-10′-nitro-2′,3′,4′,5′-tetrahydro-1′*H*-spiro[indoline-3,9′-pyrido[1,2-*a*][1,3]diazepine]-2,7′(8′*H*)-dione (5f)

Light pink powder; yield: 0.25 g, (75%); mp: 300–302 °C; ^1^H NMR (300 MHz, DMSO): *δ* = 1.78–2.10 (4H, m, 2CH_2_), 2.31 (1H, *d*, ^2^*J*_HH_ = 15.3 Hz, CH_2_CO), 3.13 (3H, s, N–CH_3_), 3.31 (1H, d, ^2^*J*_HH_ = 15.3 Hz, CH_2_CO), 3.64–3.84 (3H, m, 2CH_2_–N), 4.41–4.50 (1H, m, CH_2_N), 6.88–7.03 (3H, m, ArH), 7.26–7.31 (1H, m, ArH), 11.32 (1H, s, NH); ^13^C NMR (75 MHz, DMSO-*d*_6_): *δ* = 24.2, 24.5 (2CH_2_), 27.0 (N–CH_3_), 41.4 (**C**H_2_CO), 45.1 (CH_2_NH), 45.7 (CH_2_N), 48.0 (C_spiro_), 109.2 (C–NO_2_), 111.3, 122.4, 122.6 (4CH of Ar), 129.3, 143.7 (C–Ar), 158.5 (**C**=C–NO_2_), 167.8 (N–**C**=O), 175.5 (CH_2_–**C**=O). Anal. calcd for C_15_H_14_N_4_O_4_ (324.35): C, 59.64; H, 5.30; N, 16.37. Found C, 59.4; H, 5.6; N, 16.10.

#### 1-Ethyl-10′-nitro-2′,3′,4′,5′-tetrahydro-1′*H*-spiro[indoline-3,9′-pyrido[1,2-*a*][1,3]diazepine]-2,7′(8′*H*)-dione (5g)

Light pink powder; yield: 0.25 g, (69%); mp: 302–304 °C; ^1^H NMR (300 MHz, DMSO): *δ* = 1.13 (3H, t, *J*_HH_ = 6.9, N–CH_2_–**CH**_**3**_), 1.79–1.83 (3H, m, CH_2_), 2.03–2.19 (1H, m, CH_2_), 2.26 (1H, *d*, ^2^*J*_HH_ = 15.3 Hz, CH_2_CO), 3.32 (2H, m, N–**CH**_**2**_–CH_3_), 3.62–3.77 (4H, m, CH_2_N), 4.41–4.50 (1H, m, CH_2_N), 6.88–6.96 (2H, m, ArH), 7.06 (1H, d, *J*_HH_ = 7.8 Hz, ArH), 7.28 (1H, t, *J*_HH_ = 7.5 Hz, ArH), 11.32 (1H, s, NH); ^13^C NMR (75 MHz, DMSO-*d*_6_): *δ* = 12.1 (N–CH_2_–**C**H_3_), 24.2, 24.6 (2CH_2_), 34.8 (N–**C**H_2_–CH_3_), 41.4 (**C**H_2_CO), 45.1 (CH_2_NH), 45.7 (CH_2_N), 47.9 (C_spiro_), 109.1 (C–NO_2_), 111.3, 122.4, 122.6, 129.3, (4CH of Ar), 129.5, 142.6 (C–Ar), 158.5 (**C**=C–NO_2_), 167.8 (N–**C**=O), 175.1 (CH_2_–**C**=O). Anal. calcd for C_18_H_20_N_4_O_4_ (356.38): C, 60.66; H, 5.66; N, 15.7. Found C, 60.9; H, 5.3; N, 15.50.

The integrals in the ^1^H NMR spectrum of 5h and the particular carbon signals in the ^13^C NMR spectrum represent two structures that cannot be separated. The spectral information is given for one structure (A):

#### 10′-nitro-2′,3′,4′,5′-tetrahydro-1′*H*-spiro[indoline-3,9′-pyrido[1,2-*a*][1,3]diazepine]-2,7′(8′*H*)-dione (5h)

Pink powder; yield: 0.24 g, (72%); mp: 315–317 °C; ^1^H NMR (300 MHz, DMSO): *δ* = 1.00–1.05, 1.85–1.94 (4H, m, 2CH_2_), 2.33 (1H, *d*, ^2^*J*_HH_ = 16.2 Hz, CH_2_CO), 3.27–3.32 (2H, m, 2CH_2_NH), 3.44 (1H, d, ^2^*J*_HH_ = 16.2 Hz, CH_2_CO), 3.76 = 3.84 (2H, m, CH_2_N), 6.79–7.20 (4H, m, ArH), 9.99 (1H, s, NH), 10.20 (1H, br, NH); ^13^C NMR (75 MHz, DMSO-*d*_6_): *δ* = 25.8, 26.8 (2CH_2_), 35.7 (CH_2_NH), 36.6 (CH_2_N), 42.2 (**C**H_2_CO), 49.9 (C_spiro_), 107.2 (C–NO_2_), 116.1, 120.1, 122.6, 125.6 (4CH of Ar), 128.3, 139.4 (C–Ar), 158.1 (**C**=C–NO_2_), 167.6 (N–**C**=O), 175.0 (CH_2_–**C**=O).

The presence of two chiral centers in 5i and 5j compounds led to four isomers: (3*R*,3′*S*), (3*S*,3′*S*) and their enantiomers. The ^1^H NMR and ^13^C NMR spectra also confirmed the existence of two types of diastereoisomers.

#### 3-Methyl-8-nitro-2,3-dihydro-1*H*-spiro[imidazo[1,2-*a*]pyridine-7,3′-indoline]-2′,5(6*H*)-dione (5i)

White powder; yield: 0.25 g, (80%); mp: 301–303 °C; ^1^H NMR (300 MHz, DMSO): *δ* = 1.31–1.50 (3H, m, CH_3_), 2.32–2.48 (2H, m, CH_2_CO), 2.91–3.68 (2H, m, CH_2_NH), 3.95–4.61 (1H, m, CHMe), 6.81–6.89 (2H, m, ArH), 7.04–9.91 (2H, m, ArH), 9.91 (1H, br, NH), 10.51 (1H, s, NH); ^13^C NMR (75 MHz, DMSO-*d*_6_): *δ* = 18.4 (NH–C–**C**H_3_), 41.8 (CH_2_CO), 49.3 (C_spiro_), 49.8 (CH_2_NH), 52.3 (**C**HMe), 105.3 (C–NO_2_), 110.0, 121.9, 122.6, 128.8 (4CH of Ar), 131.9, 142.1 (C–Ar), 152.9 (**C**=C–NO_2_), 165.6 (N–**C**=O), 177.6 (CH_2_–**C**=O); MS (EI, 70 eV): *m*/*z* (%) = 314 (82) [M]^+^, 314 (59), 269 (25), 253 (100), 240 (30), 212 (33), 155 (24), 41 (25). Anal. calcd for C_15_H_14_N_4_O_4_ (314.1): C, 57.32; H, 4.49; N, 17.83.

#### 1′,3-Dimethyl-8-nitro-2,3-dihydro-1*H*-spiro[imidazo[1,2-*a*]pyridine-7,3′-indoline]-2′,5(6*H*)-dione (5j)

White powder; yield: 0.25 g, (76%); mp: 278–280 °C; ^1^H NMR (300 MHz, DMSO): *δ* = 1.34–1.40 (3H, m, CH_3_), 2.30–2.39 (1H, m, CH_2_CO), 3.01–3.11 (1H, m, CH_2_CO), 3.16 (3H, s, N–CH_3_), 3.40–3.63 (2H, m, CH_2_NH), 3.90–4.35 (1H, m, CHMe), 6.93–7.03 (2H, m, ArH), 7.17 (1H, t, ^3^*J*_HH_ = 6.9 Hz, ArH), 7.29 (1H, t, ^3^*J*_HH_ = 7.8 Hz, ArH), 9.82 (1H, br, NH), 9.95 (1H, br, NH); ^13^C NMR (75 MHz, DMSO-*d*_6_): *δ* = 20.1 (C–CH_3_), 26.9 (N–CH_3_), 41.6 (CH_2_CO), 49.2 (C_spiro_), 49.9 (CH_2_NH), 52.3 (**C**HMe), 105.2 (C–NO_2_), 109.05, 122.4, 122.6, 129.1 (4CH of Ar), 131.0, 143.6 (C–Ar), 152.8 (**C**=C–NO_2_), 165.7 (N–C=O), 175.9 (CH_2_–**C**=O). Anal. calcd for C_16_H_16_N_4_O_4_ (328.32): C, 58.53; H, 4.91; N, 17.06. Found C, 58.2; H, 4.6; N, 17.30.

## Conclusion

We designed a novel and convenient procedure for the synthesis of three new classes of spiropyridineoxindoles with fused heterocyclic compounds (imidazole, pyrimidine and diazepine) in good yields *via* a four-component reaction among 1,1-bis(methylthio)-2-nitroethylene, various aliphatic diamines, Meldrum's acid and isatin derivatives using a catalytic amount of *p*-TSA. The present process has several important features including mild and facile reaction conditions, easy accessibility of reactants, a simple workup procedure, the use of ethanol as a solvent, short reaction times, and good-to-high yields. These structures having both indole and fused-pyridine moieties, which are some of the most typical privileged scaffolds, are completely new and there is no other report on their synthesis.

## Conflicts of interest

The authors declare no competing financial interest.

## Supplementary Material

RA-009-C8RA10379H-s001

## References

[cit1] Da-Silva J. F. M., Garden S. J., Pinto A. C. (2001). J. Braz. Chem. Soc..

[cit2] Abdel-Rahman A. H., Keshk E. M., Hanna M. A., El-Bady S. M. (2006). Bioorg. Med. Chem..

[cit3] Kang T. H., Matsumoto K., Murakami Y., Takayama H., Kitajima M., Aimi N., Watanabe H. (2002). Eur. J. Pharmacol..

[cit4] Usui T., Kondoh M., Cui C.-B., Mayumi T., Osada H. (1998). Biochem. J..

[cit5] Thakor S. F., Dinesh M., Patel M. P., Patel R. G. (2007). Saudi Pharm. J..

[cit6] Vintonyak V. V., Warburg K., Kruse H., Grimme S., Hubel K., Rauth D., Waldmann H. (2010). Angew. Chem., Int. Ed..

[cit7] Gatta F., Pomponi M., Marta M. (1991). J. Heterocycl. Chem..

[cit8] Kumari G., Nutan M., Modi M., Gupta S. K., Singh R. K. (2011). Eur. J. Med. Chem..

[cit9] Thangamani A. (2010). Eur. J. Med. Chem..

[cit10] Rosenmond P., Hosseini-Merescht M., Bub C. (1994). Liebigs Ann. Chem..

[cit11] Ding K., Lu Y., Nikolovska-Coleska Z., Qui S., Ding Y., Gao W., Stuckey J., Krajewski K. (2004). J. Am. Chem. Soc..

[cit12] Okita T., Isobe M. (1994). Tetrahedron.

[cit13] Yeung B. K. S., Zou B., Rottmann M., Lakshminarayana S. B., Ang S. H., Leong S. Y., Tan J., Wong J., Fischli C., Winzeler E. A., Petersen F., Brun R., Dartois V., Diagana T. T. (2010). J. Med. Chem..

[cit14] Chen T., Xu X. P., Ji S. J. (2010). J. Comb. Chem..

[cit15] Yan S. J., Niu Y.-F., Huang R., Lin J. (2009). Synlett..

[cit16] Meia G. J., Shi F. (2018). Chem. Commun..

[cit17] Rahmati A., Khalesi Z. (2012). Tetrahedron.

[cit18] Stucchi M., Lesma G., Meneghetti F., Rainoldi G., Sacchetti A., Silvani A. (2016). J. Org. Chem..

[cit19] Alizadeh A., Moafi L. (2015). Helv. Chim. Acta.

[cit20] Nagalakshmi R. A., Suresh J., Sivakumar S., Ranjith Kumar R., Nilantha Lakshman P. L. (2014). Acta Crystallogr..

[cit21] Yu F., Huang R., Ni H., Fan J., Yan S., Lin J. (2013). Green Chem..

[cit22] Ghadiri S., Bayat M., Hosseini F. (2019). Monatsh. Chem..

