# Supplementary material for: Synthesis of spiroimidazopyridineoxindole, spiropyridopyrimidineoxindole and spiropyridodiazepineoxindole derivatives based on heterocyclic ketene aminals via a four-component reaction
Source: RSC Adv. 2019 May 24;9(29):16384–9. doi: 10.1039/c8ra10379h (PMC9064345; doi:10.1039/c8ra10379h)
Supplement: RA-009-C8RA10379H-s001 [file RA-009-C8RA10379H-s001.pdf]

Electronic Supplementary Material (ESI) for RSC Advances.
This journal is © The Royal Society of Chemistry 2019

Supporting Information

Synthesis of Spiroimidazopyridineoxindole, Spiropyridopyrimidineoxindole and Spiropyridodiazepineoxindole Structures Based on Heterocyclic Ketene Aminals *via* a Four-Component Reaction

Fatemeh Rahimi, Mohammad Bayat*, Hajar Hosseini

Chemistry Department, Imam Khomeini International University, Qazvin, Iran

e-mail: bayat_mo@yahoo.com, m.bayat@sci.ikiu.ac.ir)

The Table of Contents

Title	Page
Title, author's name, address and table of contents	1
Experimental Section; General remarks	2
**Figure 1.** The ^{1}H and ^{13}C NMR chemical shifts of **5a**	2
**Figure 2.** Structure of all products **5**	3
^{1}H and ^{13}C NMR and IR and Mass spectrums of **5a**	4-7
^{1}H and ^{13}C NMR and IR and Mass spectrums of **5b**	8-11
^{1}H and D_2O exchange and ^{13}C NMR and IR and Mass spectrums of **5c**	12-16
^{1}H and D_2O exchange and ^{13}C NMR (in DMSO and $CDCl_3$) and IR spectrums of **5d**	17-20
^{1}H and ^{13}C NMR and Mass spectrums of **5e**	21-23
^{1}H and ^{13}C NMR spectrums of **5f**	24-25
^{1}H and ^{13}C NMR spectrums of **5g**	26-27
^{1}H and ^{13}C NMR spectrums of **5h**	28-29
^{1}H and expand and ^{13}C NMR spectrums of **5i**	30-32
^{1}H and ^{13}C NMR spectrums of **5j**	33-34

Experimental Section

General remarks:

Melting points were measured on an Electrothermal 9100 apparatus. Mass spectra were recorded with an Agilent 5975C VL MSD with Triple-Axis Detector operating at an ionization potential of 70 Ev. ^{1}H and ^{13}C NMR spectra were measured (DMSO) with a Bruker DRX-300 AVANCE spectrometer at 300 and 75 MHz, respectively. IR spectra were recorded on a Bruker Tensor 27, ῡ in cm^{-1}. All NMR spectra at room temperature were determined in DMSO-d_6. Chemical shifts are reported in parts per million (δ) downfield from an internal tetramethylsilane reference. Coupling constants (J values) are reported in hertz (Hz), and spin multiplicities are indicated by the following symbols: s (singlet), d (doublet), t (triplet), q (quartet), m (multiplet). All chemicals were purchased from Merck or Aldrich and were used without further purification.

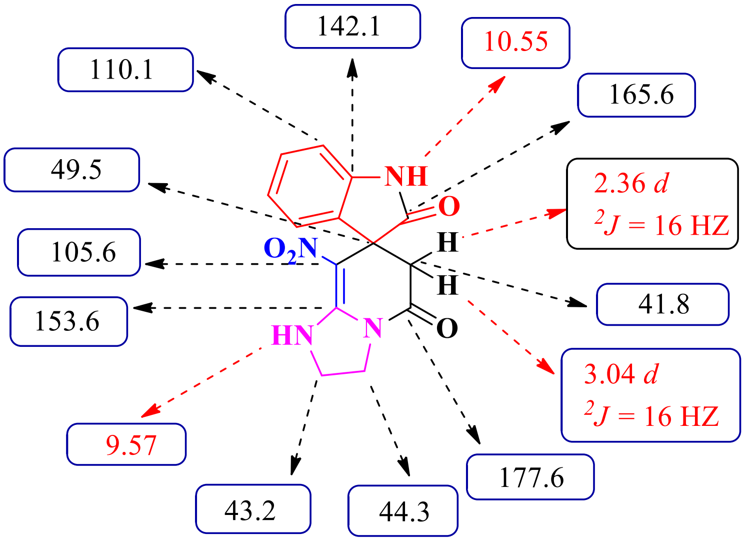

Figure 1. The ^{1}H and ^{13}C NMR chemical shifts of **5a.**

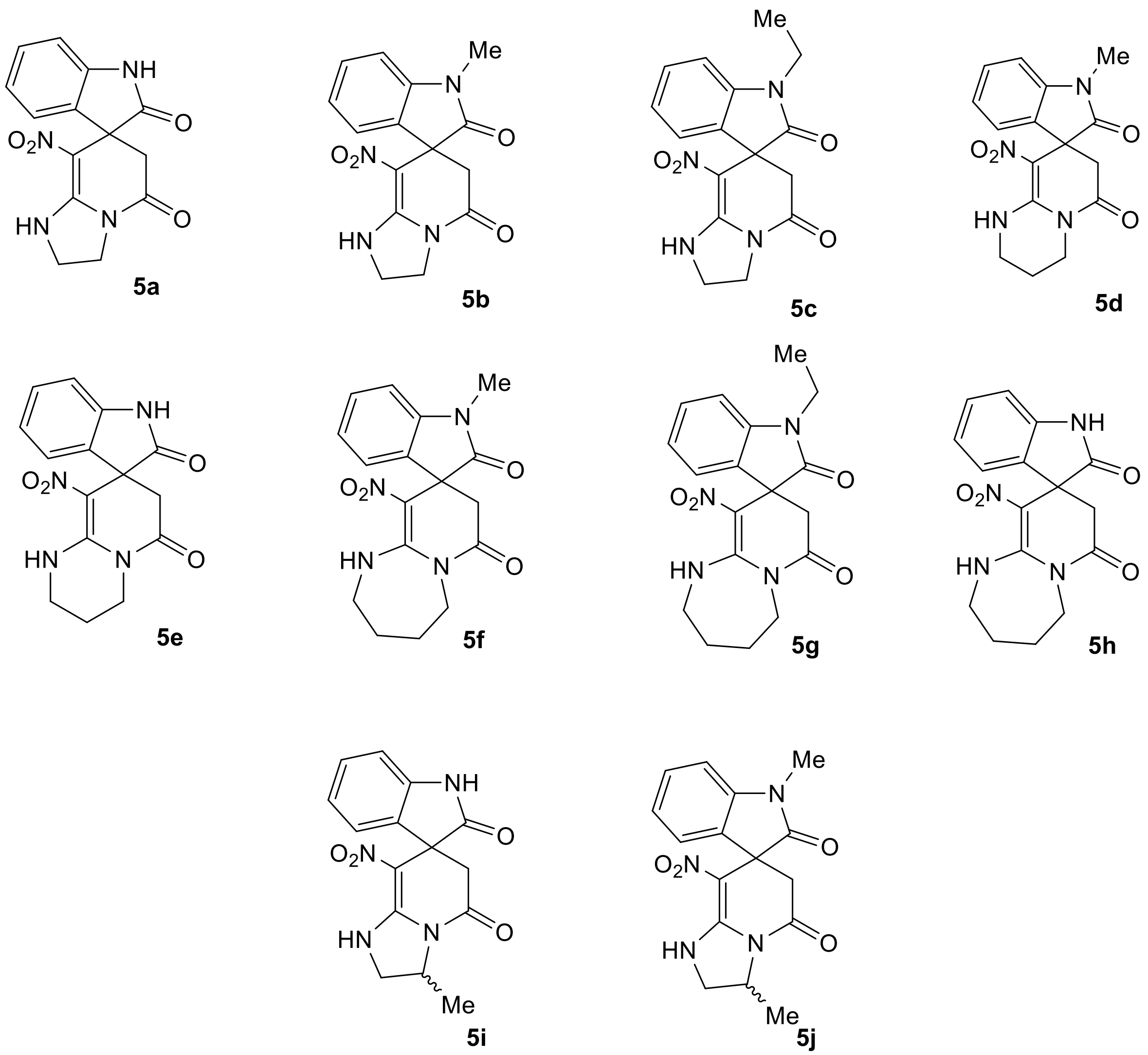

Figure 2. Structure of all products **5**

The structures of all products **5a-j** were deduced from their IR, mass, ^{1}H NMR, and ^{13}C NMR spectra.

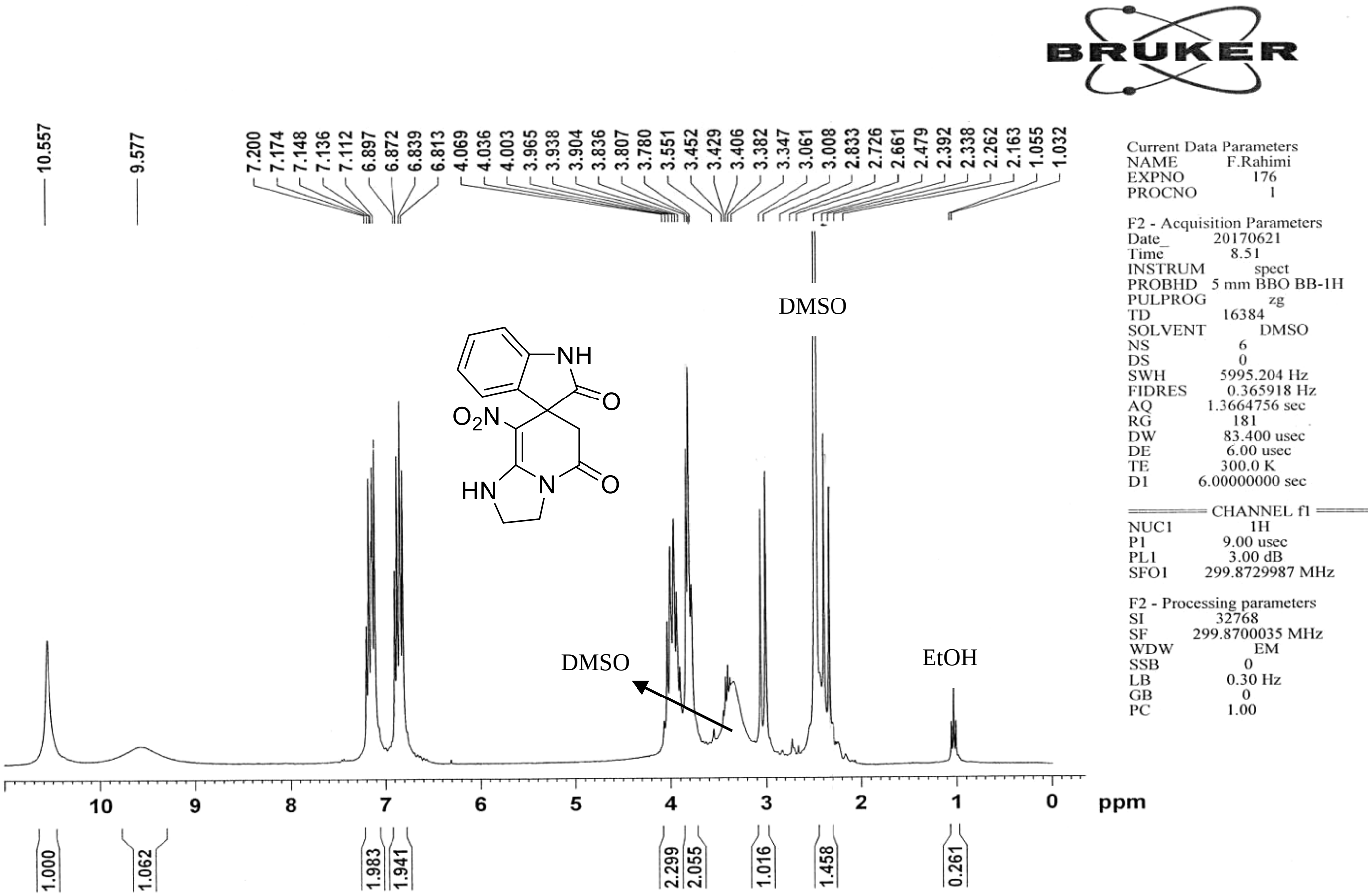
BRUKER
10.557
9.577
7.200
7.174
7.148
7.136
7.112
6.897
6.872
6.839
6.813
4.069
4.036
4.003
3.965
3.938
3.904
3.836
3.807
3.780
3.551
3.452
3.429
3.406
3.382
3.347
3.061
3.008
2.833
2.726
2.661
2.479
2.392
2.338
2.262
2.163
1.055
1.032
DMSO
NH
O
O_2N
HN
N
O
DMSO
EtOH
ppm
1.000
1.062
1.983
1.941
2.299
2.055
1.016
1.458
0.261
Current Data Parameters
NAME F.Rahimi
EXPNO 176
PROCNO 1
F2 - Acquisition Parameters
Date_ 20170621
Time 8.51
INSTRUM spect
PROBHD 5 mm BBO BB-1H
PULPROG zg
TD 16384
SOLVENT DMSO
NS 6
DS 0
SWH 5995.204 Hz
FIDRES 0.365918 Hz
AQ 1.3664756 sec
RG 181
DW 83.400 usec
DE 6.00 usec
TE 300.0 K
D1 6.00000000 sec
CHANNEL f1
NUC1 1H
P1 9.00 usec
PL1 3.00 dB
SFO1 299.8729987 MHz
F2 - Processing parameters
SI 32768
SF 299.8700035 MHz
WDW EM
SSB 0
LB 0.30 Hz
GB 0
PC 1.00

^{1}H NMR of 5a

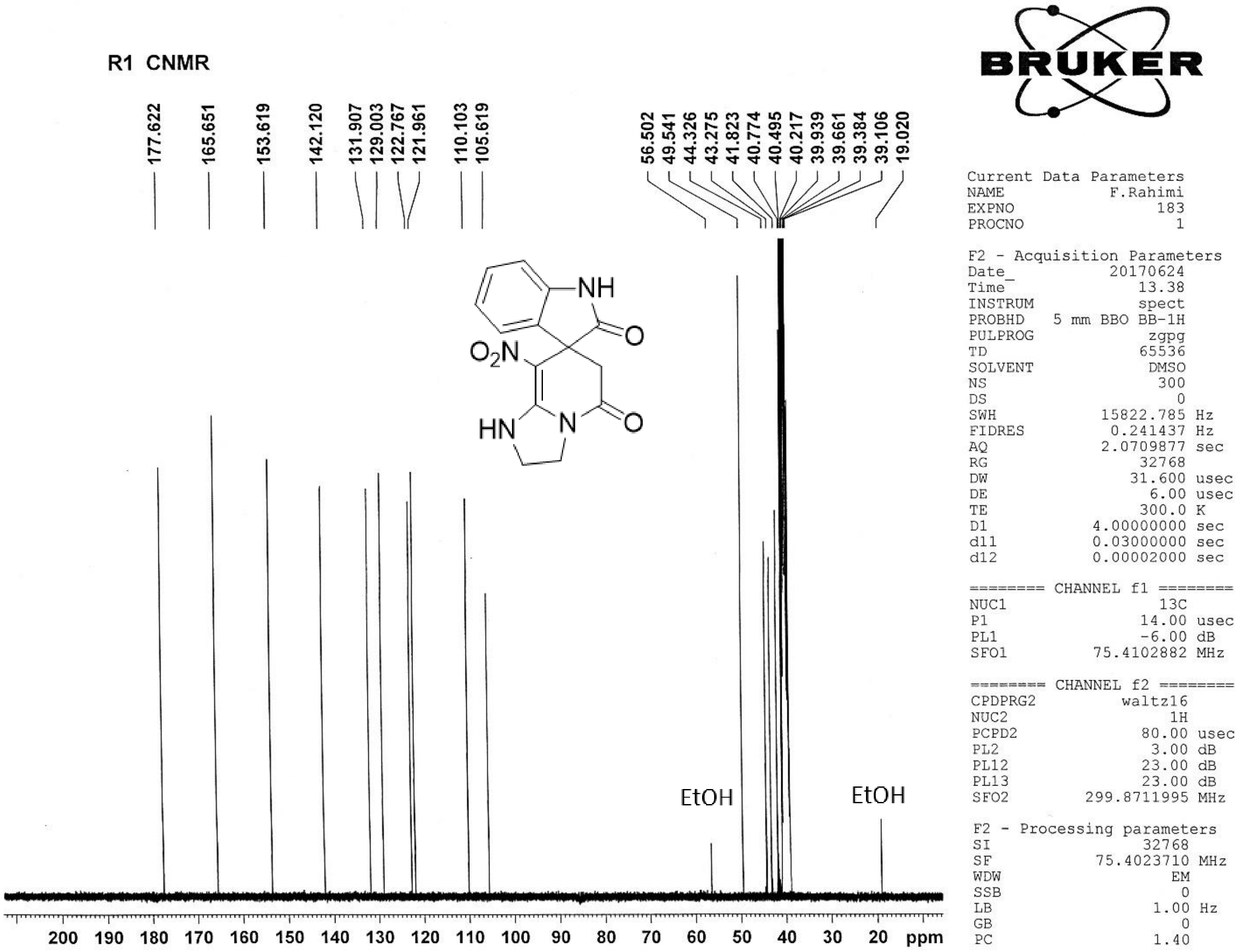
R1 CNMR
BRUKER
177.622
165.651
153.619
142.120
131.907
129.003
122.767
121.961
110.103
105.619
56.502
49.541
44.326
43.275
41.823
40.774
40.495
40.217
39.939
39.661
39.384
39.106
19.020
NH
O
O2N
HN
N
O
EtOH
EtOH
200 190 180 170 160 150 140 130 120 110 100 90 80 70 60 50 40 30 20 ppm
Current Data Parameters
NAME F.Rahimi
EXPNO 183
PROCNO 1
F2 - Acquisition Parameters
Date_ 20170624
Time 13.38
INSTRUM spect
PROBHD 5 mm BBO BB-1H
PULPROG zgpg
TD 65536
SOLVENT DMSO
NS 300
DS 0
SWH 15822.785 Hz
FIDRES 0.241437 Hz
AQ 2.0709877 sec
RG 32768
DW 31.600 usec
DE 6.00 usec
TE 300.0 K
D1 4.00000000 sec
d11 0.03000000 sec
d12 0.00002000 sec
======== CHANNEL f1 ========
NUC1 13C
P1 14.00 usec
PL1 -6.00 dB
SFO1 75.4102882 MHz
======== CHANNEL f2 ========
CPDPRG2 waltz16
NUC2 1H
PCPD2 80.00 usec
PL2 3.00 dB
PL12 23.00 dB
PL13 23.00 dB
SFO2 299.8711995 MHz
F2 - Processing parameters
SI 32768
SF 75.4023710 MHz
WDW EM
SSB 0
LB 1.00 Hz
GB 0
PC 1.40

^{13}C NMR of 5a

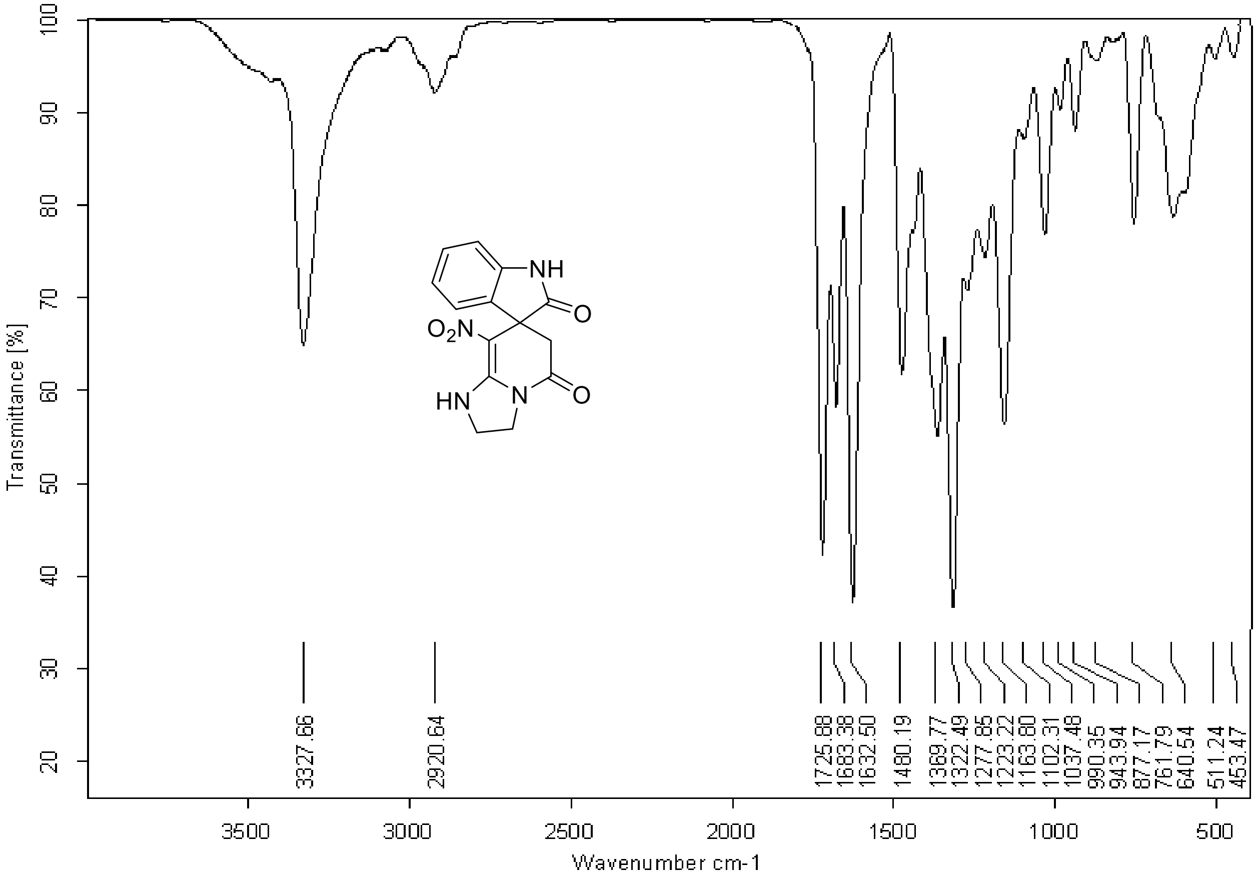

100
90
80
70
60
50
40
30
20
Transmittance [%]
3327.66
2920.64
1725.88
1683.38
1632.50
1480.19
1369.77
1322.49
1277.85
1223.22
1163.80
1102.31
1037.48
990.35
943.94
877.17
761.79
640.54
511.24
453.47
3500
3000
2500
2000
1500
1000
500
Wavenumber cm-1
NH
O
O2N
HN
N
O

IR of 5a

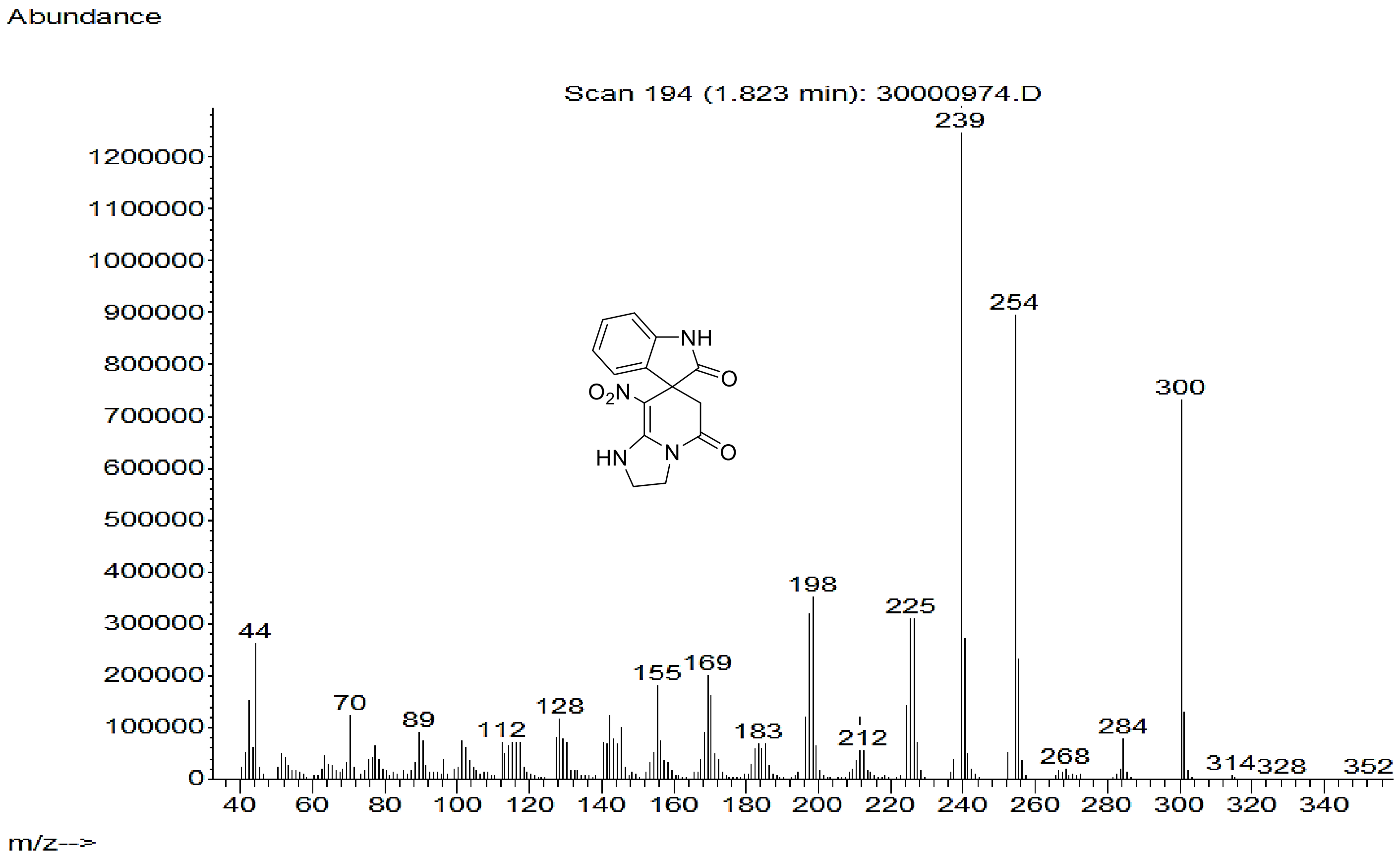
Abundance
Scan 194 (1.823 min): 30000974.D
1200000
1100000
1000000
900000
800000
700000
600000
500000
400000
300000
200000
100000
0
239
254
300
198
225
44
155
169
70
89
112
128
183
212
284
268
314
328
352
40 60 80 100 120 140 160 180 200 220 240 260 280 300 320 340
m/z-->
NH
O
O_2N
HN
N
O

Mass of 5a

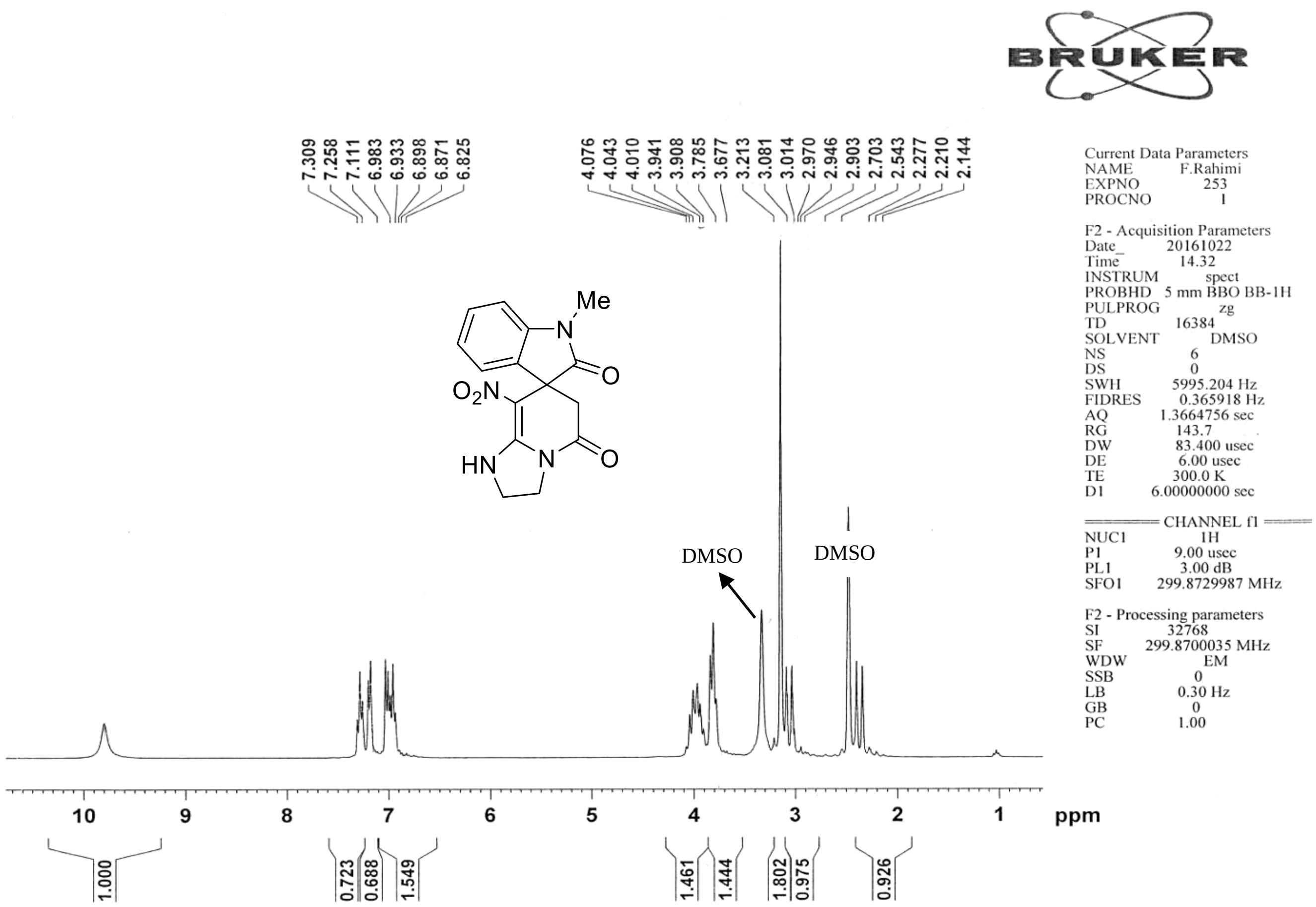

7.309
7.258
7.111
6.983
6.933
6.898
6.871
6.825
4.076
4.043
4.010
3.941
3.908
3.785
3.677
3.213
3.081
3.014
2.970
2.946
2.903
2.703
2.543
2.277
2.210
2.144
Me
N
O
O_2N
HN
N
O
DMSO
DMSO
Current Data Parameters
NAME F.Rahimi
EXPNO 253
PROCNO 1
F2 - Acquisition Parameters
Date_ 20161022
Time 14.32
INSTRUM spect
PROBHD 5 mm BBO BB-1H
PULPROG zg
TD 16384
SOLVENT DMSO
NS 6
DS 0
SWH 5995.204 Hz
FIDRES 0.365918 Hz
AQ 1.3664756 sec
RG 143.7
DW 83.400 usec
DE 6.00 usec
TE 300.0 K
D1 6.00000000 sec
CHANNEL f1
NUC1 1H
P1 9.00 usec
PL1 3.00 dB
SFO1 299.8729987 MHz
F2 - Processing parameters
SI 32768
SF 299.8700035 MHz
WDW EM
SSB 0
LB 0.30 Hz
GB 0
PC 1.00
10 9 8 7 6 5 4 3 2 1 ppm
1.000
0.723
0.688
1.549
1.461
1.444
1.802
0.975
0.926

^{1}H NMR of 5b

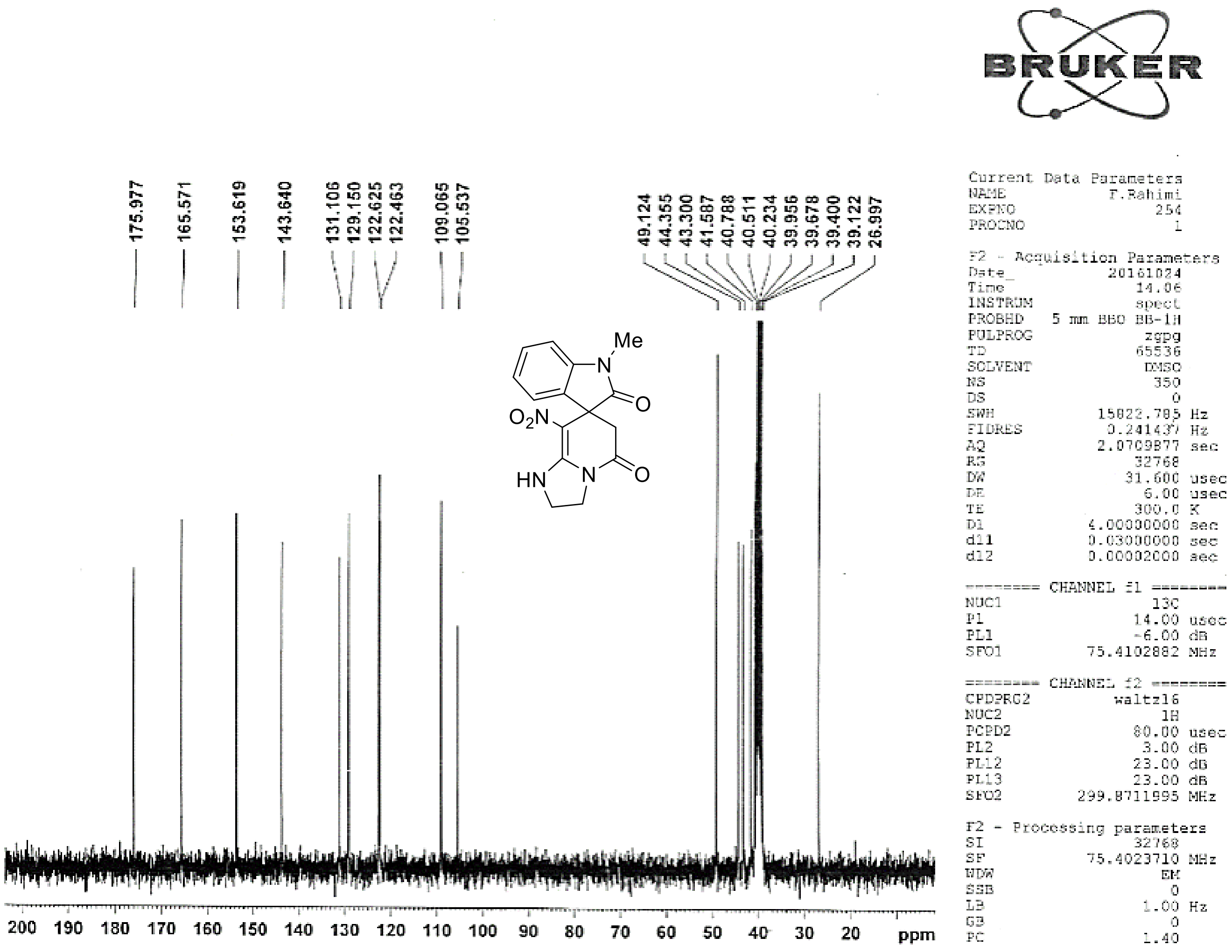

^{13}C NMR of 5b

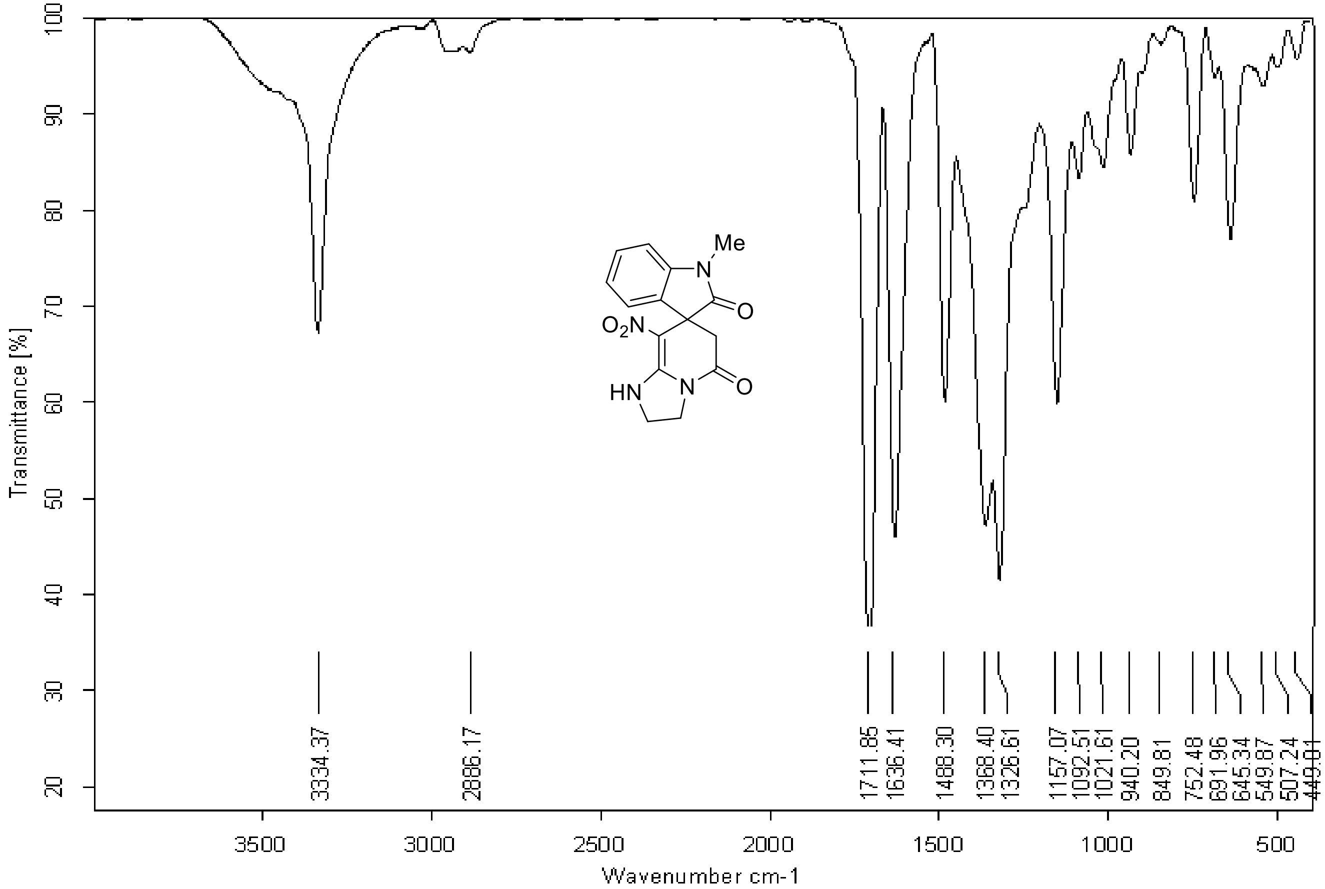

100
90
80
70
60
50
40
30
20
Transmittance [%]
3334.37
2886.17
1711.85
1636.41
1488.30
1368.40
1326.61
1157.07
1092.51
1021.61
940.20
849.81
752.48
691.96
645.34
549.87
507.24
449.01
3500
3000
2500
2000
1500
1000
500
Wavenumber cm-1
Me
N
O
O_2N
HN
N
O

IR of 5b

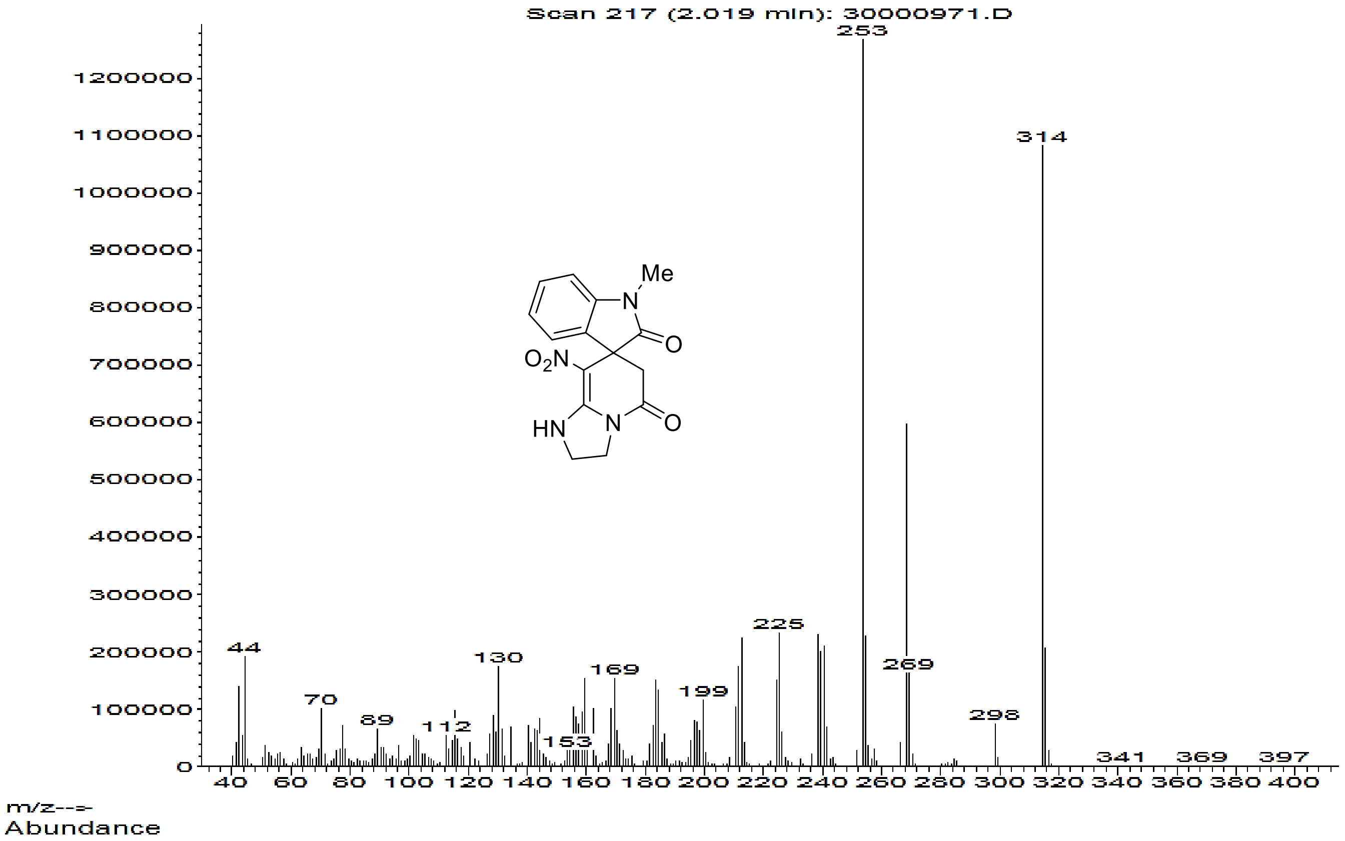
Scan 217 (2.019 min): 30000971.D
Abundance
m/z-->
253
314
269
225
169
199
130
44
70
89
112
153
298
341
369
397
Me
N
O
O₂N
HN
N
O

Mass of 5b

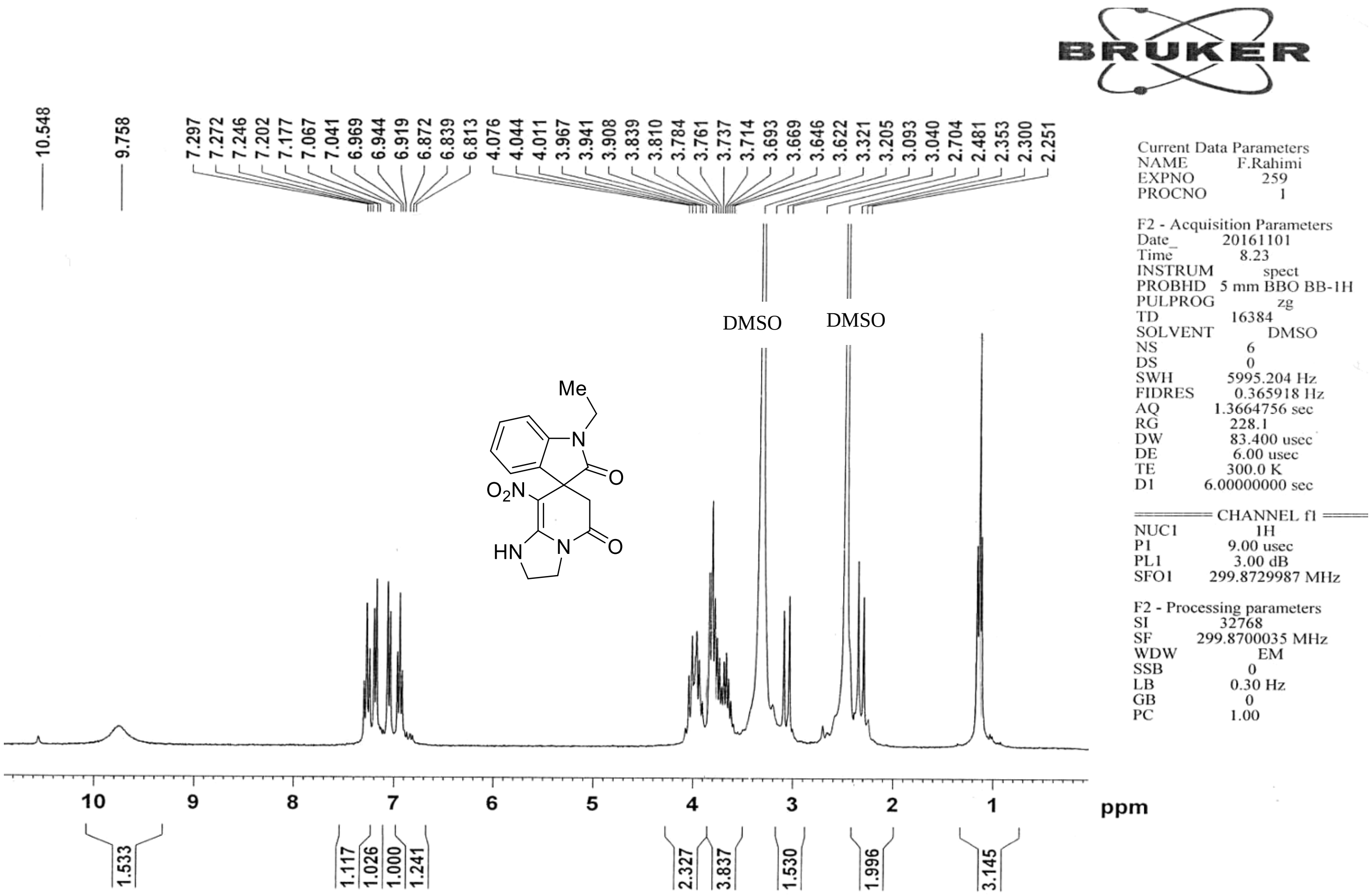
BRUKER
10.548
9.758
7.297
7.272
7.246
7.202
7.177
7.067
7.041
6.969
6.944
6.919
6.872
6.839
6.813
4.076
4.044
4.011
3.967
3.941
3.908
3.839
3.810
3.784
3.761
3.737
3.714
3.693
3.669
3.646
3.622
3.321
3.205
3.093
3.040
2.704
2.481
2.353
2.300
2.251
DMSO
DMSO
Me
N
O
O_2N
O
HN
N
Current Data Parameters
NAME F.Rahimi
EXPNO 259
PROCNO 1
F2 - Acquisition Parameters
Date_ 20161101
Time 8.23
INSTRUM spect
PROBHD 5 mm BBO BB-1H
PULPROG zg
TD 16384
SOLVENT DMSO
NS 6
DS 0
SWH 5995.204 Hz
FIDRES 0.365918 Hz
AQ 1.3664756 sec
RG 228.1
DW 83.400 usec
DE 6.00 usec
TE 300.0 K
D1 6.00000000 sec
CHANNEL f1
NUC1 1H
P1 9.00 usec
PL1 3.00 dB
SFO1 299.8729987 MHz
F2 - Processing parameters
SI 32768
SF 299.8700035 MHz
WDW EM
SSB 0
LB 0.30 Hz
GB 0
PC 1.00
10
9
8
7
6
5
4
3
2
1
ppm
1.533
1.117
1.026
1.000
1.241
2.327
3.837
1.530
1.996
3.145

^{1}H NMR of 5c

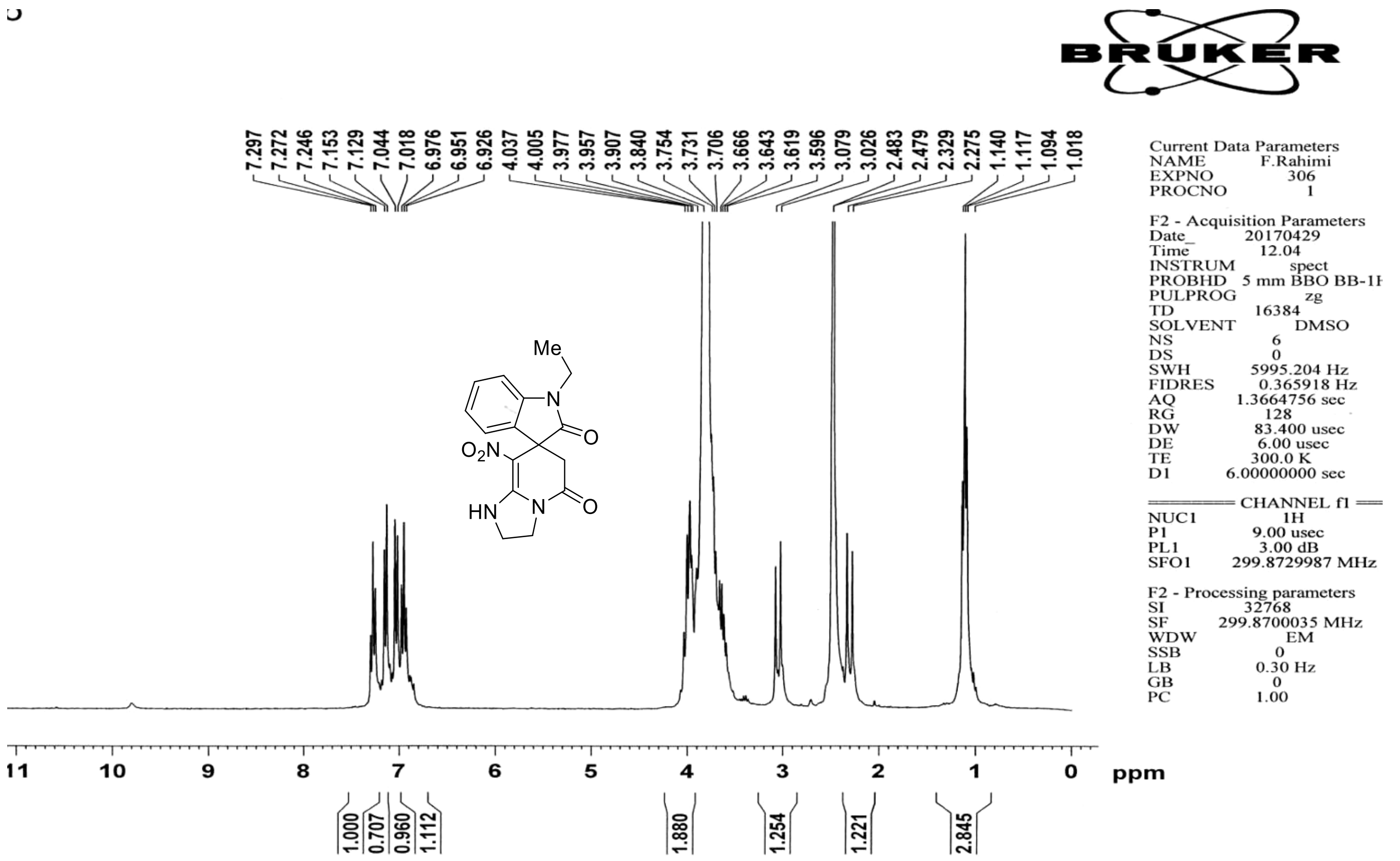

^{1}H NMR with D_2O of 5c

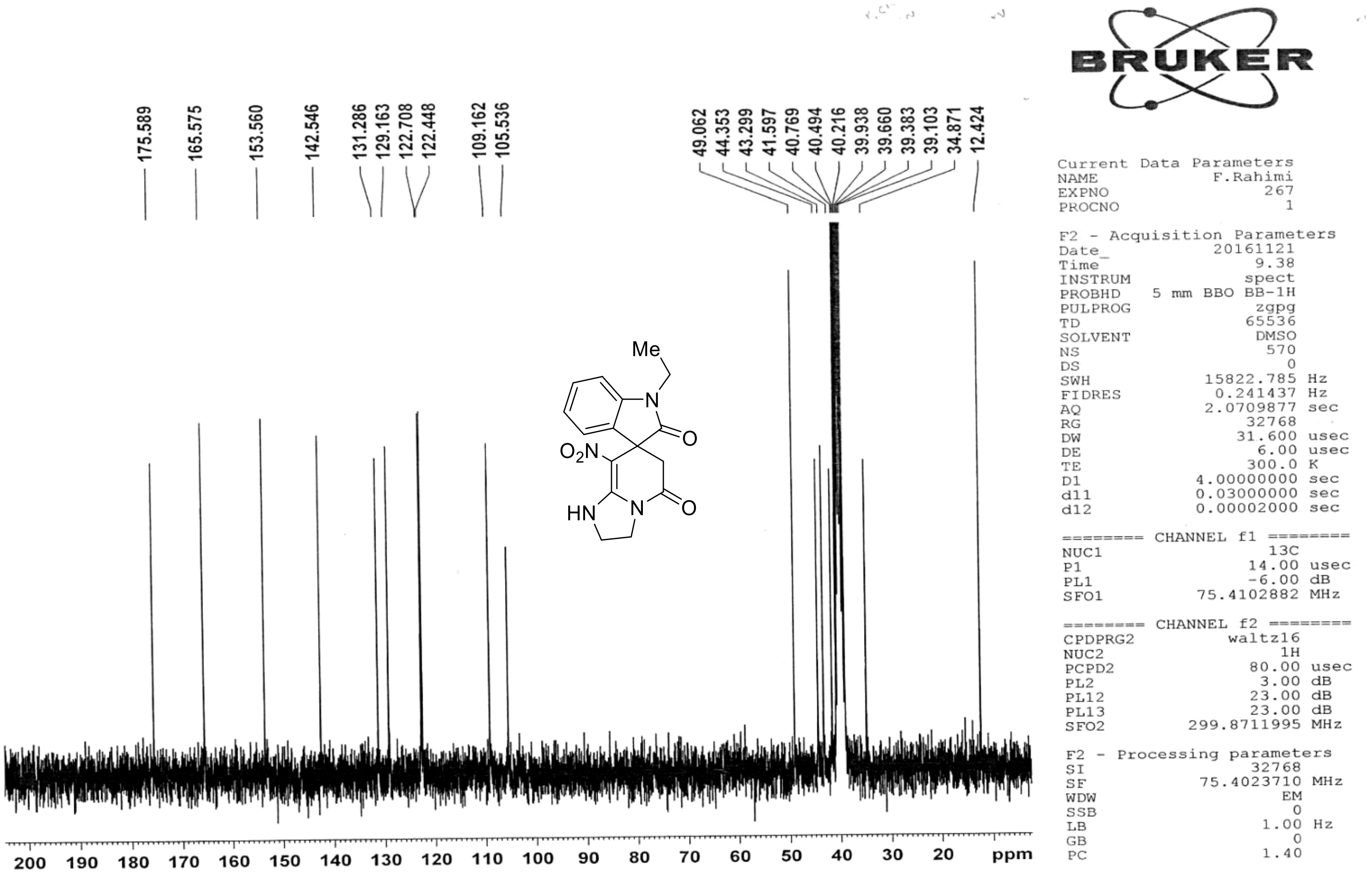

175.589
165.575
153.560
142.546
131.286
129.163
122.708
122.448
109.162
105.536
49.062
44.353
43.299
41.597
40.769
40.494
40.216
39.938
39.660
39.383
39.103
34.871
12.424
BRUKER
Me
N
O
O_2N
HN
N
O
200 190 180 170 160 150 140 130 120 110 100 90 80 70 60 50 40 30 20 ppm
Current Data Parameters
NAME F.Rahimi
EXPNO 267
PROCNO 1
F2 - Acquisition Parameters
Date_ 20161121
Time 9.38
INSTRUM spect
PROBHD 5 mm BBO BB-1H
PULPROG zgpg
TD 65536
SOLVENT DMSO
NS 570
DS 0
SWH 15822.785 Hz
FIDRES 0.241437 Hz
AQ 2.0709877 sec
RG 32768
DW 31.600 usec
DE 6.00 usec
TE 300.0 K
D1 4.00000000 sec
d11 0.03000000 sec
d12 0.00002000 sec
======== CHANNEL f1 ========
NUC1 13C
P1 14.00 usec
PL1 -6.00 dB
SFO1 75.4102882 MHz
======== CHANNEL f2 ========
CPDPRG2 waltz16
NUC2 1H
PCPD2 80.00 usec
PL2 3.00 dB
PL12 23.00 dB
PL13 23.00 dB
SFO2 299.8711995 MHz
F2 - Processing parameters
SI 32768
SF 75.4023710 MHz
WDW EM
SSB 0
LB 1.00 Hz
GB 0
PC 1.40

^{13}C NMR of 5c

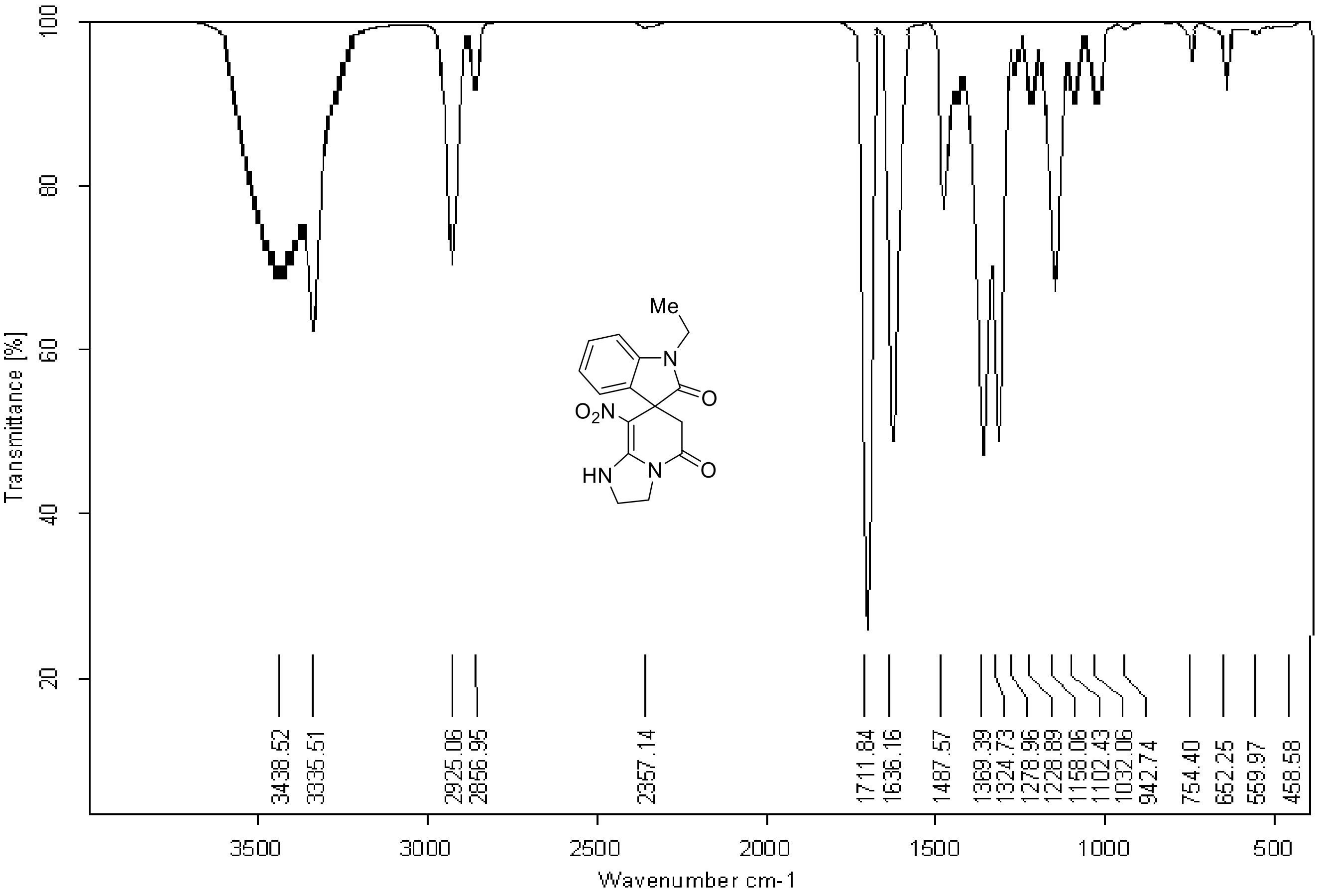

Transmittance [%]
100
80
60
40
20
Wavenumber cm-1
3500
3000
2500
2000
1500
1000
500
3438.52
3335.51
2925.06
2856.95
2357.14
1711.84
1636.16
1487.57
1369.39
1324.73
1278.96
1228.89
1158.06
1102.43
1032.06
942.74
754.40
652.25
559.97
458.58
Me
N
O
O_2N
HN
N
O

IR of 5c

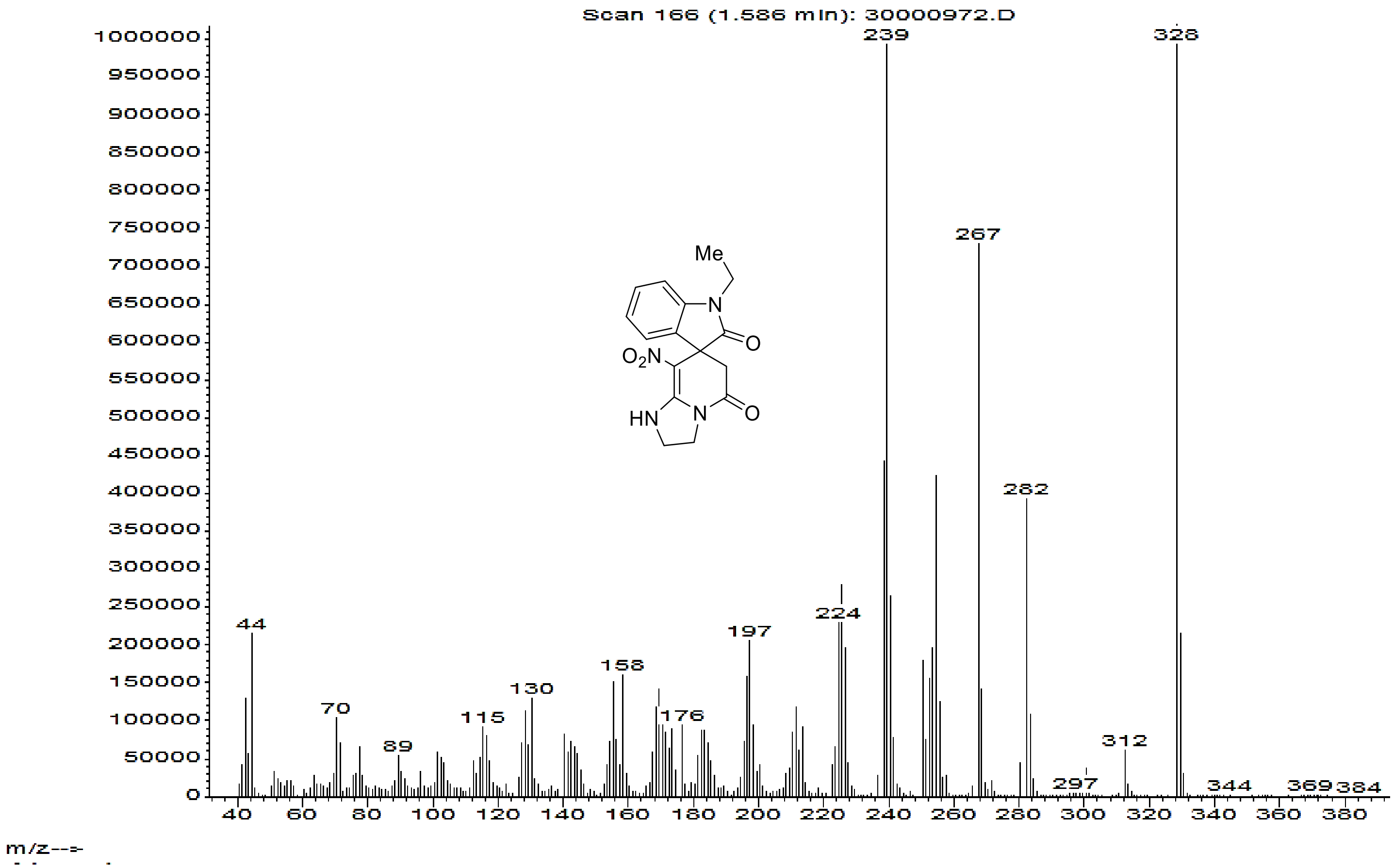
Scan 166 (1.586 min): 30000972.D
m/z-->
Me
N
O
O2N
HN
N
O
44
70
89
115
130
158
176
197
224
239
267
282
297
312
328
344
369
384

Mass of 6c

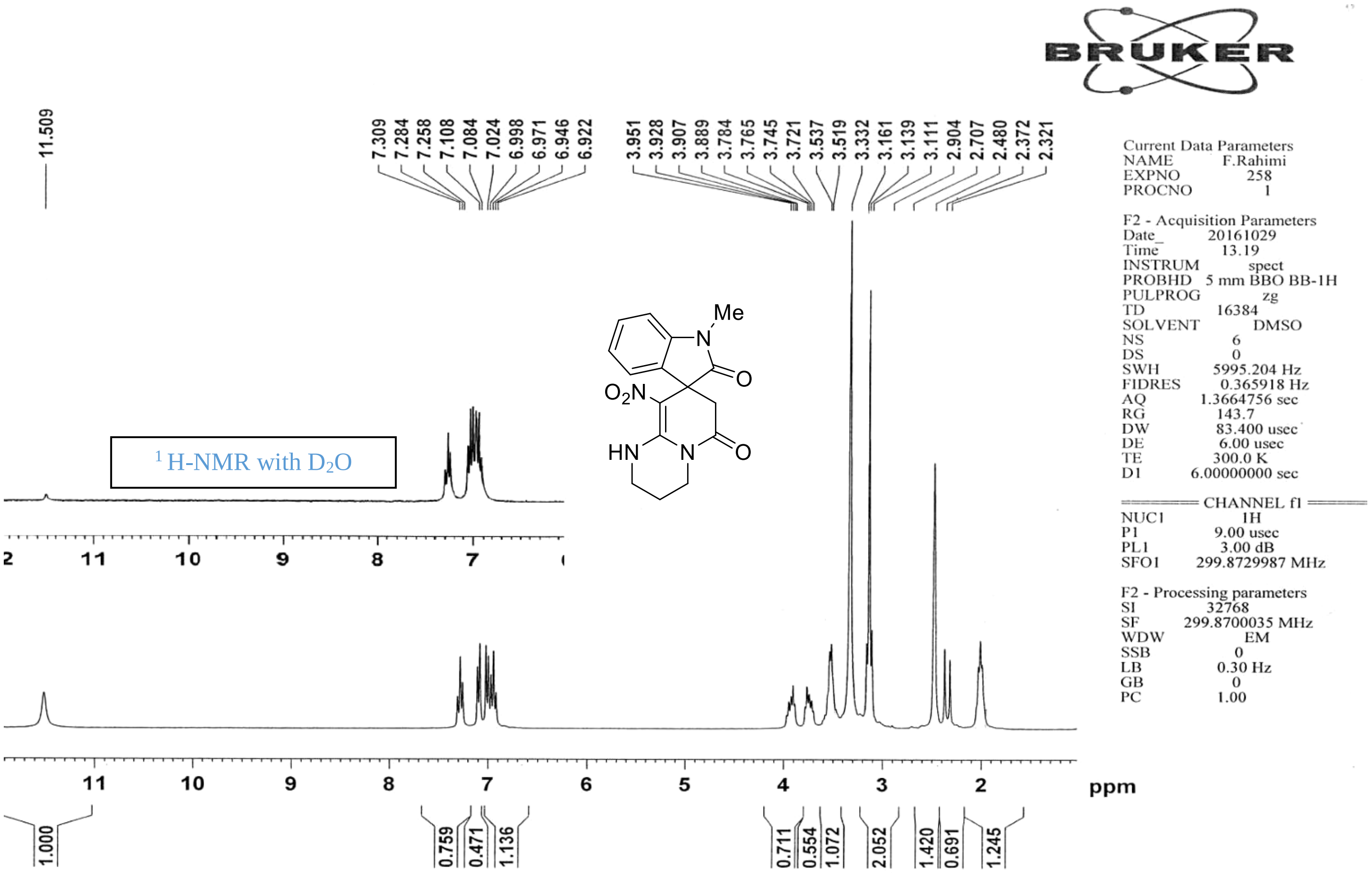
BRUKER
1H-NMR with D2O
Me
N
O
O2N
HN
N
O
11.509
7.309
7.284
7.258
7.108
7.084
7.024
6.998
6.971
6.946
6.922
3.951
3.928
3.907
3.889
3.784
3.765
3.745
3.721
3.537
3.519
3.332
3.161
3.139
3.111
2.904
2.707
2.480
2.372
2.321
ppm
1.000
0.759
0.471
1.136
0.711
0.554
1.072
2.052
1.420
0.691
1.245
Current Data Parameters
NAME F.Rahimi
EXPNO 258
PROCNO 1
F2 - Acquisition Parameters
Date_ 20161029
Time 13.19
INSTRUM spect
PROBHD 5 mm BBO BB-1H
PULPROG zg
TD 16384
SOLVENT DMSO
NS 6
DS 0
SWH 5995.204 Hz
FIDRES 0.365918 Hz
AQ 1.3664756 sec
RG 143.7
DW 83.400 usec
DE 6.00 usec
TE 300.0 K
D1 6.00000000 sec
CHANNEL f1
NUC1 1H
P1 9.00 usec
PL1 3.00 dB
SFO1 299.8729987 MHz
F2 - Processing parameters
SI 32768
SF 299.8700035 MHz
WDW EM
SSB 0
LB 0.30 Hz
GB 0
PC 1.00

^{1}H NMR of 5d

In the spectrum taken in DMSO, three carbon signals are lost, but when the chloroform was used (which solves product **5d** well), the three signals appeared (two spectrums below):

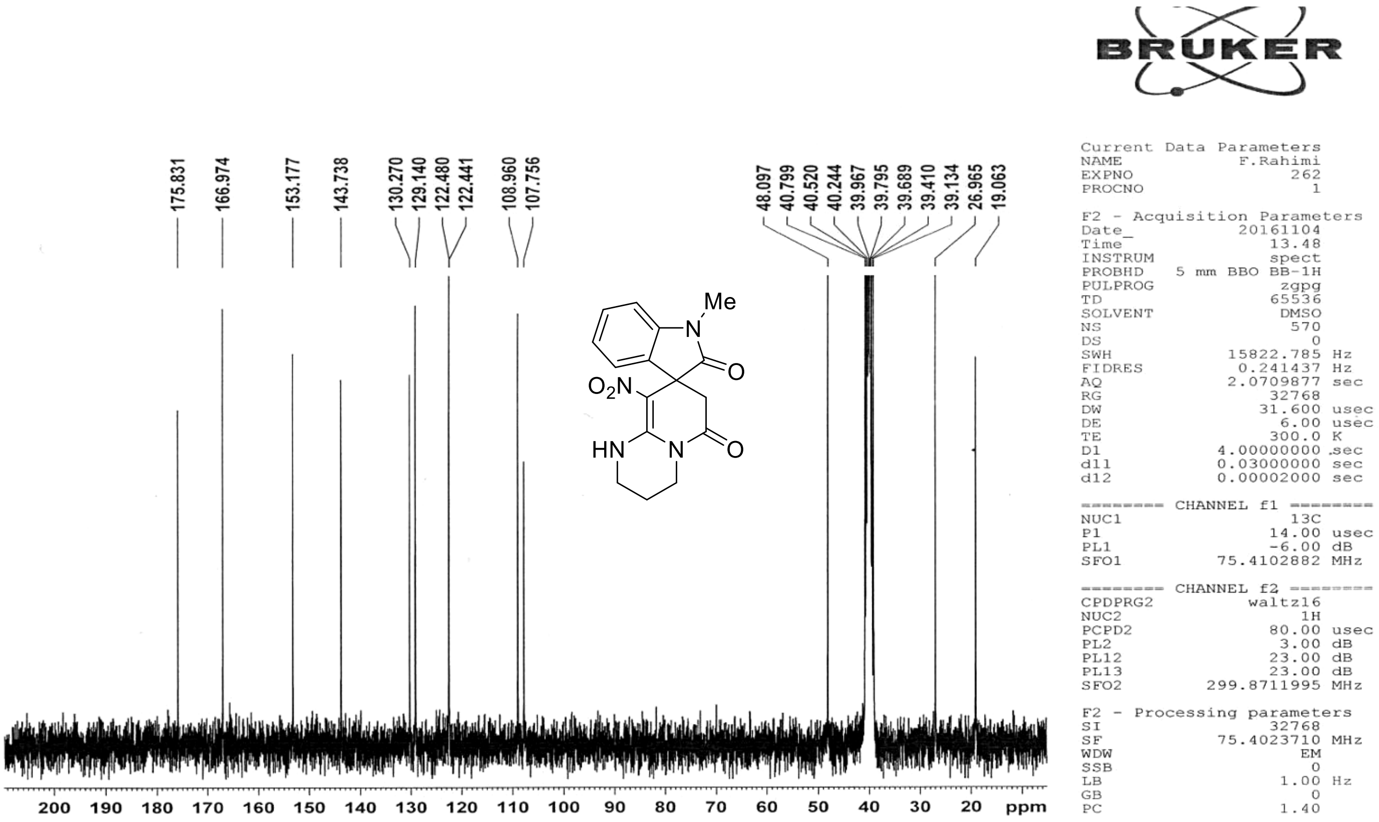

^{13}C NMR of 5d (in DMSO)

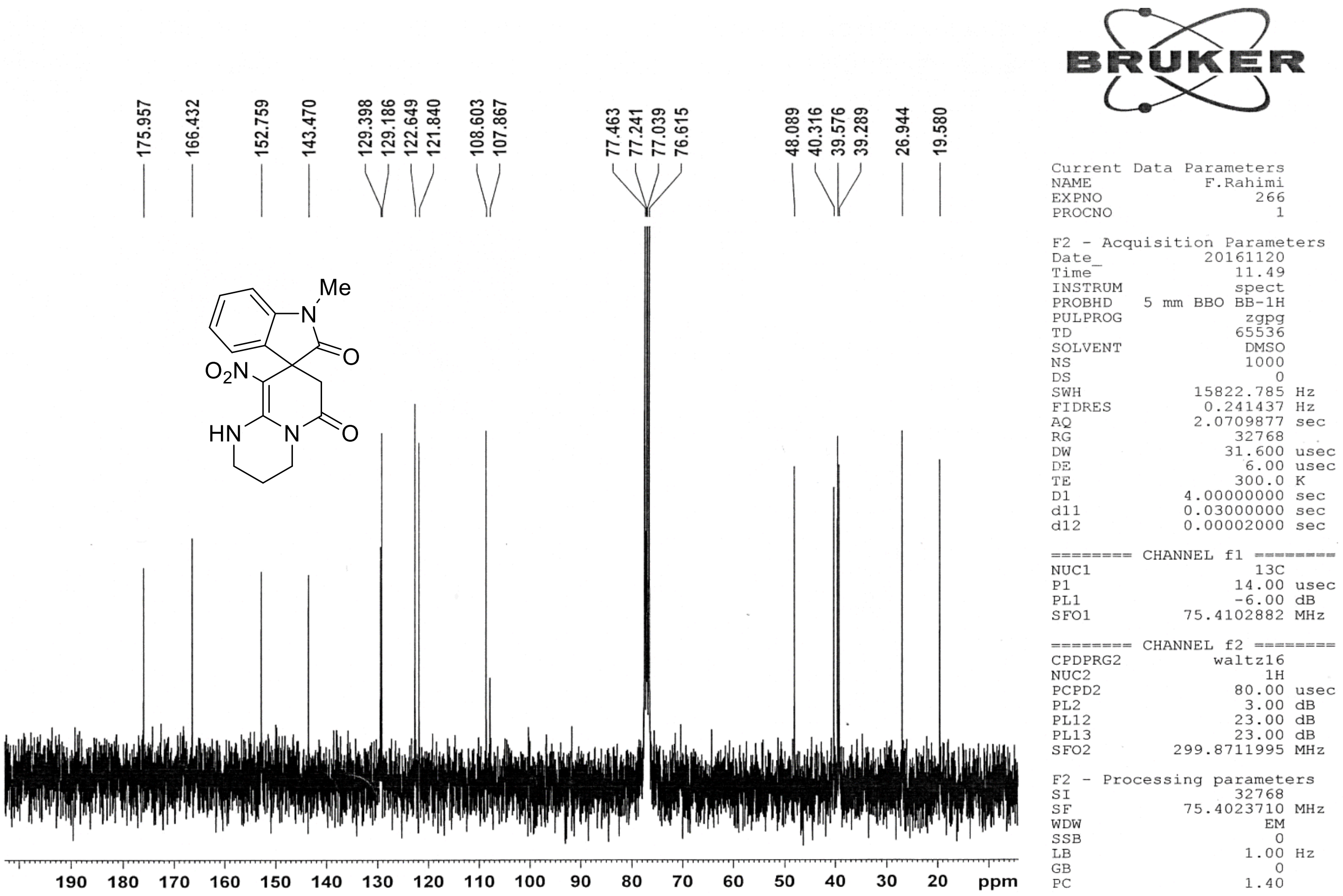

^{13}C NMR of 5d (in $CDCl_3$)

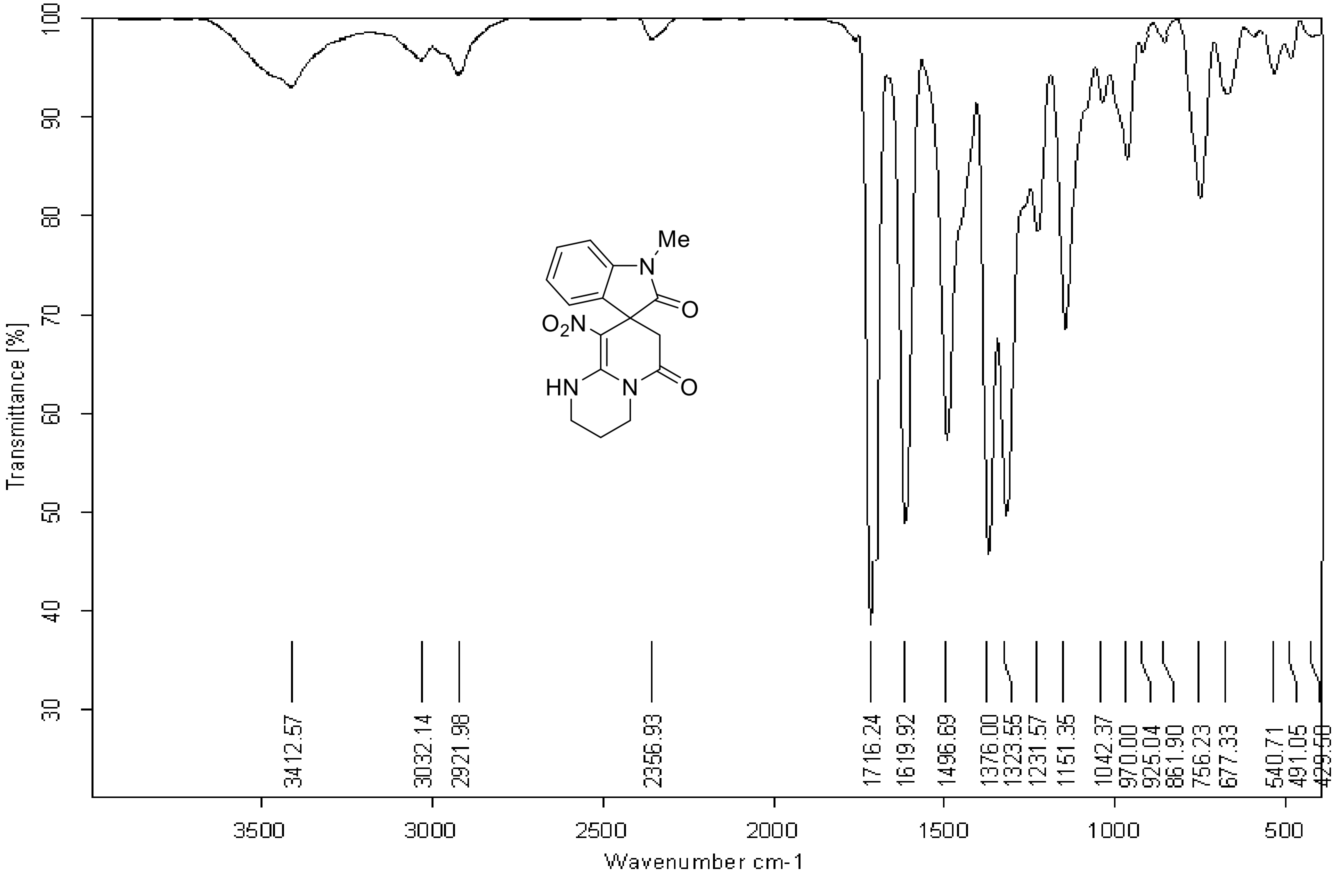
Transmittance [%]
Wavenumber cm-1
Me
N
O
O_2N
HN
N
O
3412.57
3032.14
2921.98
2356.93
1716.24
1619.92
1496.69
1376.00
1323.55
1231.57
1151.35
1042.37
970.00
925.04
861.90
756.23
677.33
540.71
491.05
429.50

IR of 5d

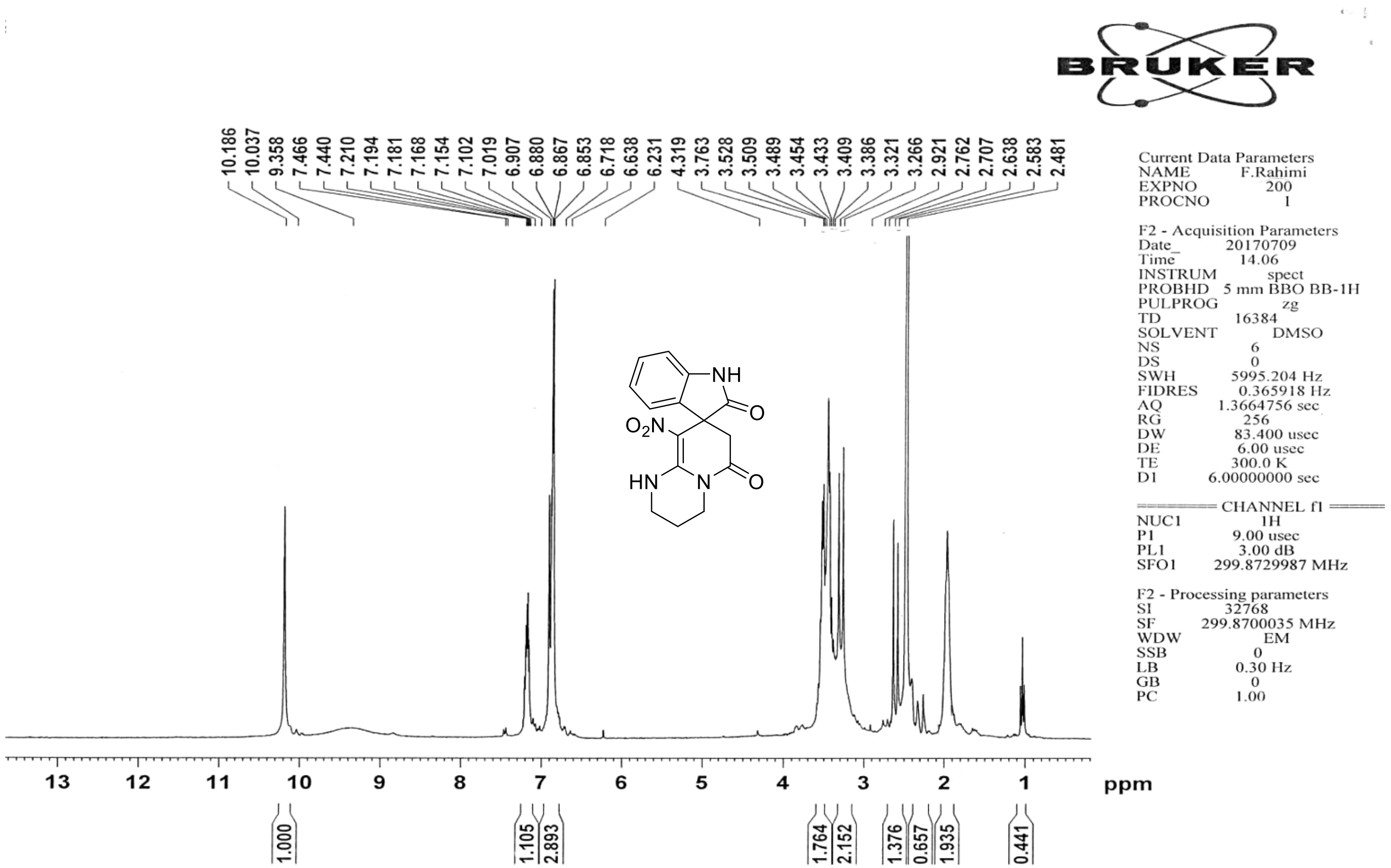

BRUKER
10.186
10.037
9.358
7.466
7.440
7.210
7.194
7.181
7.168
7.154
7.102
7.019
6.907
6.880
6.867
6.853
6.718
6.638
6.231
4.319
3.763
3.528
3.509
3.489
3.454
3.433
3.409
3.386
3.321
3.266
2.921
2.762
2.707
2.638
2.583
2.481
NH
O
O_2N
HN
N
O
Current Data Parameters
NAME F.Rahimi
EXPNO 200
PROCNO 1
F2 - Acquisition Parameters
Date_ 20170709
Time 14.06
INSTRUM spect
PROBHD 5 mm BBO BB-1H
PULPROG zg
TD 16384
SOLVENT DMSO
NS 6
DS 0
SWH 5995.204 Hz
FIDRES 0.365918 Hz
AQ 1.3664756 sec
RG 256
DW 83.400 usec
DE 6.00 usec
TE 300.0 K
D1 6.00000000 sec
CHANNEL f1
NUC1 1H
P1 9.00 usec
PL1 3.00 dB
SFO1 299.8729987 MHz
F2 - Processing parameters
SI 32768
SF 299.8700035 MHz
WDW EM
SSB 0
LB 0.30 Hz
GB 0
PC 1.00
13
12
11
10
9
8
7
6
5
4
3
2
1
ppm
1.000
1.105
2.893
1.764
2.152
1.376
0.657
1.935
0.441

^{1}H NMR of 5e

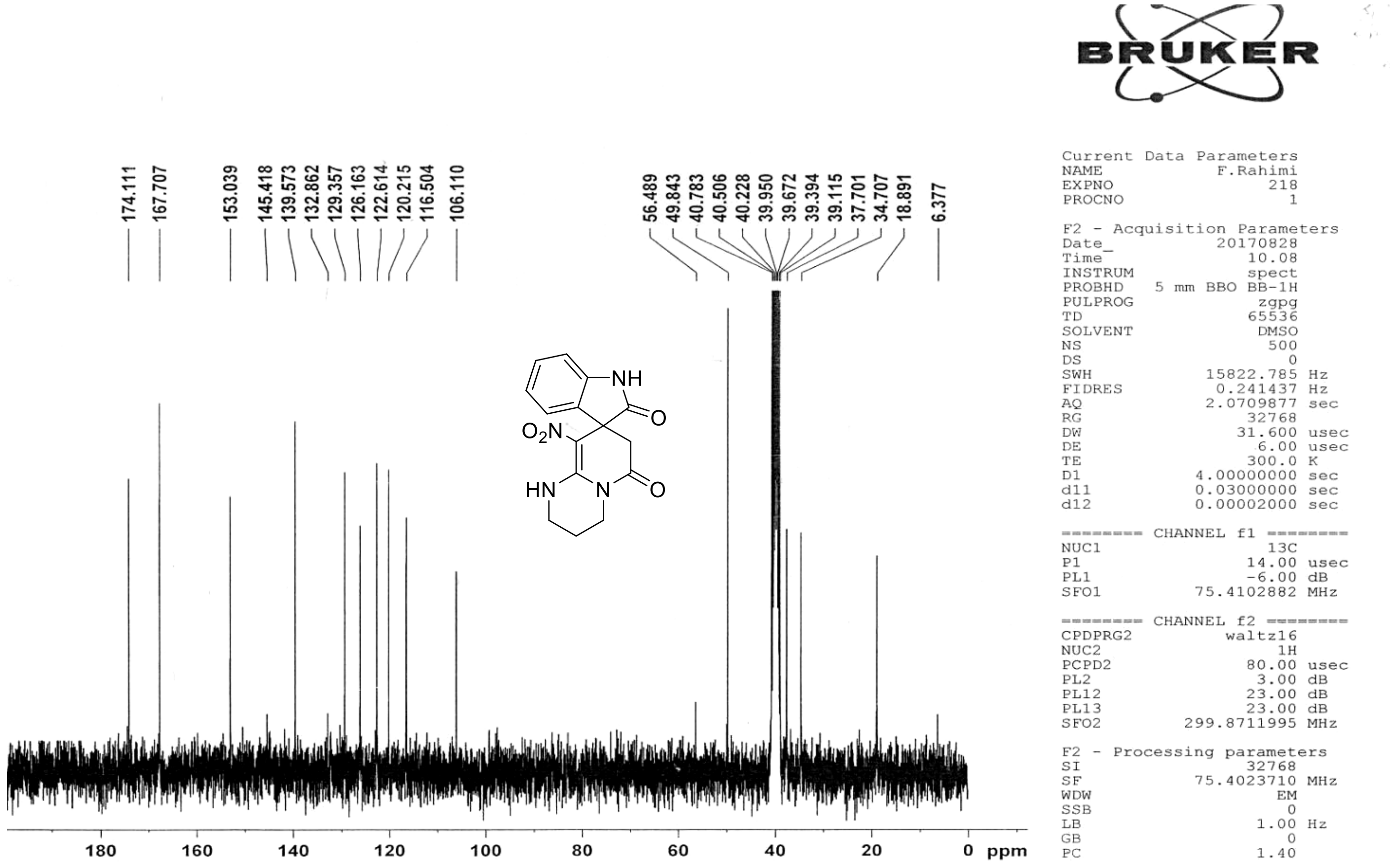

174.111
167.707
153.039
145.418
139.573
132.862
129.357
126.163
122.614
120.215
116.504
106.110
56.489
49.843
40.783
40.506
40.228
39.950
39.672
39.394
39.115
37.701
34.707
18.891
6.377
NH
O
O_2N
O
HN
N
180 160 140 120 100 80 60 40 20 0 ppm
BRUKER
Current Data Parameters
NAME F.Rahimi
EXPNO 218
PROCNO 1
F2 - Acquisition Parameters
Date_ 20170828
Time 10.08
INSTRUM spect
PROBHD 5 mm BBO BB-1H
PULPROG zgpg
TD 65536
SOLVENT DMSO
NS 500
DS 0
SWH 15822.785 Hz
FIDRES 0.241437 Hz
AQ 2.0709877 sec
RG 32768
DW 31.600 usec
DE 6.00 usec
TE 300.0 K
D1 4.00000000 sec
d11 0.03000000 sec
d12 0.00002000 sec
======== CHANNEL f1 ========
NUC1 13C
P1 14.00 usec
PL1 -6.00 dB
SFO1 75.4102882 MHz
======== CHANNEL f2 ========
CPDPRG2 waltz16
NUC2 1H
PCPD2 80.00 usec
PL2 3.00 dB
PL12 23.00 dB
PL13 23.00 dB
SFO2 299.8711995 MHz
F2 - Processing parameters
SI 32768
SF 75.4023710 MHz
WDW EM
SSB 0
LB 1.00 Hz
GB 0
PC 1.40

^{13}C NMR of 5e

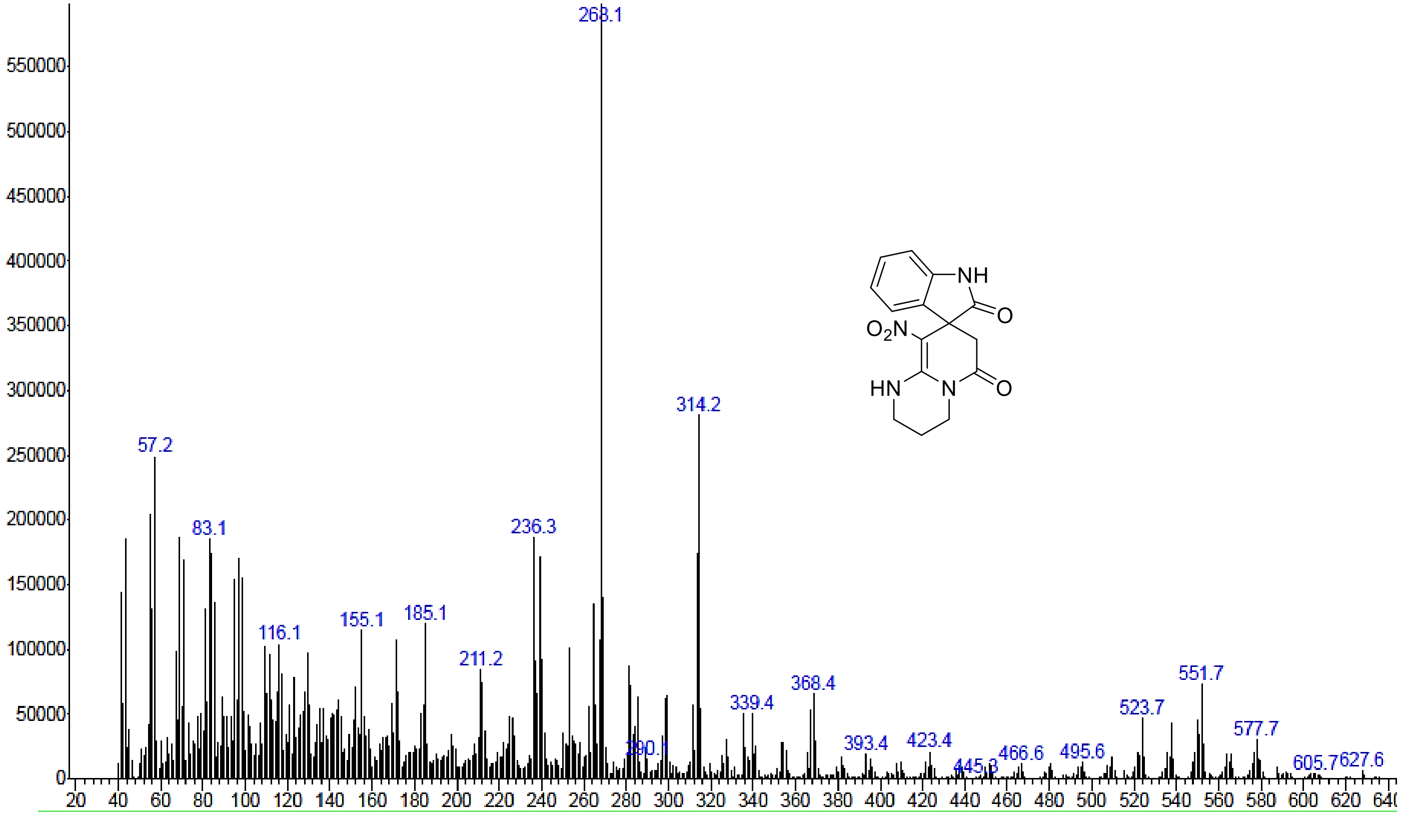
268.1
314.2
57.2
83.1
236.3
116.1
155.1
185.1
211.2
339.4
368.4
393.4
423.4
466.6
495.6
523.7
551.7
577.7
605.7
627.6
NH
O
O_2N
HN
N
O

Mass of 5e

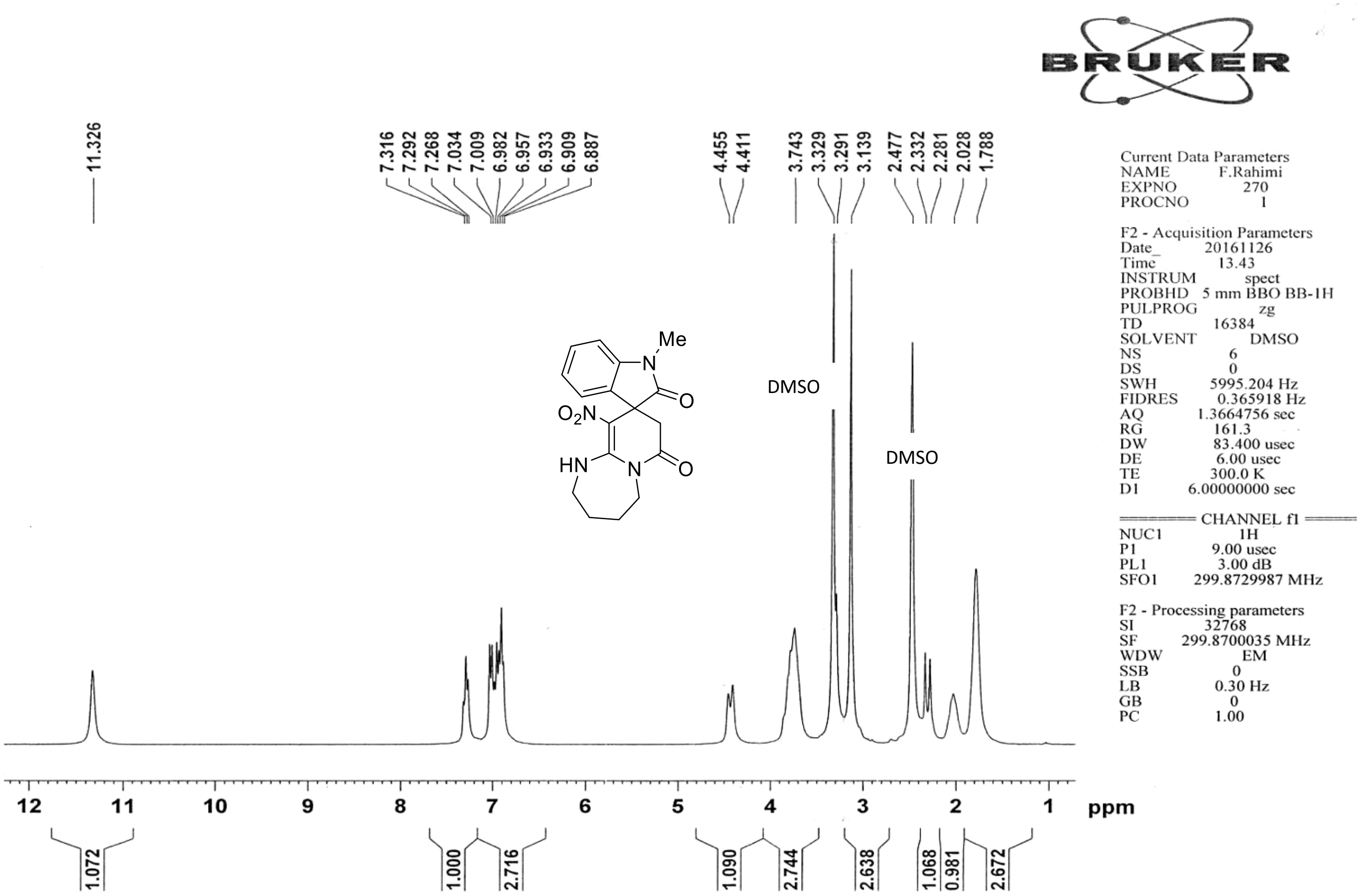
BRUKER
11.326
7.316 7.292 7.268 7.034 7.009 6.982 6.957 6.933 6.909 6.887
4.455 4.411
3.743 3.329 3.291 3.139 2.477 2.332 2.281 2.028 1.788
Me
N
O
O_2N
HN
N
O
DMSO
DMSO
Current Data Parameters
NAME F.Rahimi
EXPNO 270
PROCNO 1
F2 - Acquisition Parameters
Date_ 20161126
Time 13.43
INSTRUM spect
PROBHD 5 mm BBO BB-1H
PULPROG zg
TD 16384
SOLVENT DMSO
NS 6
DS 0
SWH 5995.204 Hz
FIDRES 0.365918 Hz
AQ 1.3664756 sec
RG 161.3
DW 83.400 usec
DE 6.00 usec
TE 300.0 K
D1 6.00000000 sec
CHANNEL f1
NUC1 1H
P1 9.00 usec
PL1 3.00 dB
SFO1 299.8729987 MHz
F2 - Processing parameters
SI 32768
SF 299.8700035 MHz
WDW EM
SSB 0
LB 0.30 Hz
GB 0
PC 1.00
12 11 10 9 8 7 6 5 4 3 2 1 ppm
1.072
1.000 2.716
1.090 2.744
2.638
1.068 0.981 2.672

^{1}H NMR of 5f

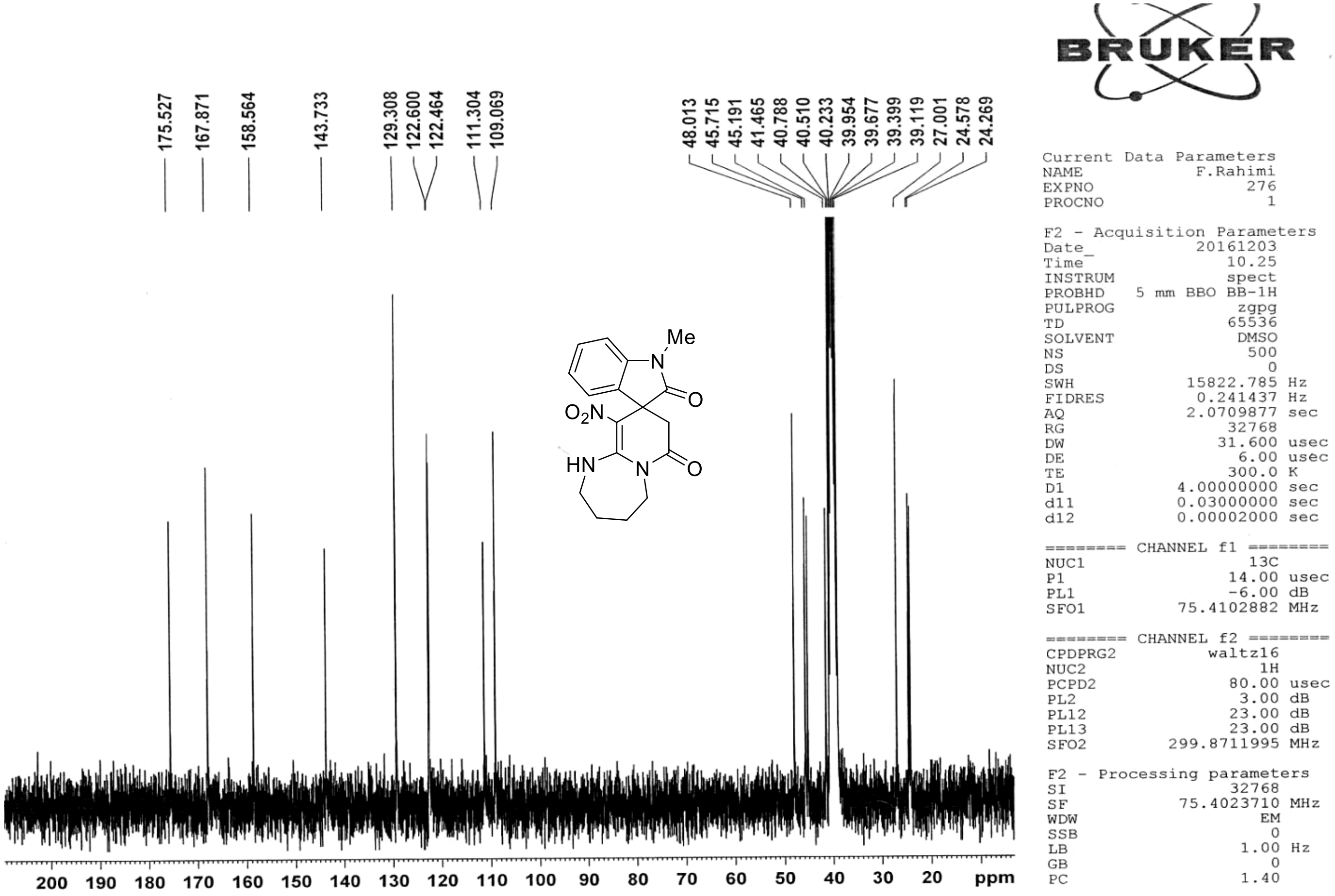
175.527
167.871
158.564
143.733
129.308
122.600
122.464
111.304
109.069
48.013
45.715
45.191
41.465
40.788
40.510
40.233
39.954
39.677
39.399
39.119
27.001
24.578
24.269
Me
N
O
O_2N
HN
N
O
BRUKER
Current Data Parameters
NAME F.Rahimi
EXPNO 276
PROCNO 1
F2 - Acquisition Parameters
Date_ 20161203
Time 10.25
INSTRUM spect
PROBHD 5 mm BBO BB-1H
PULPROG zgpg
TD 65536
SOLVENT DMSO
NS 500
DS 0
SWH 15822.785 Hz
FIDRES 0.241437 Hz
AQ 2.0709877 sec
RG 32768
DW 31.600 usec
DE 6.00 usec
TE 300.0 K
D1 4.00000000 sec
d11 0.03000000 sec
d12 0.00002000 sec
======== CHANNEL f1 ========
NUC1 13C
P1 14.00 usec
PL1 -6.00 dB
SFO1 75.4102882 MHz
======== CHANNEL f2 ========
CPDPRG2 waltz16
NUC2 1H
PCPD2 80.00 usec
PL2 3.00 dB
PL12 23.00 dB
PL13 23.00 dB
SFO2 299.8711995 MHz
F2 - Processing parameters
SI 32768
SF 75.4023710 MHz
WDW EM
SSB 0
LB 1.00 Hz
GB 0
PC 1.40
200 190 180 170 160 150 140 130 120 110 100 90 80 70 60 50 40 30 20 ppm

^{13}C NMR of 5f

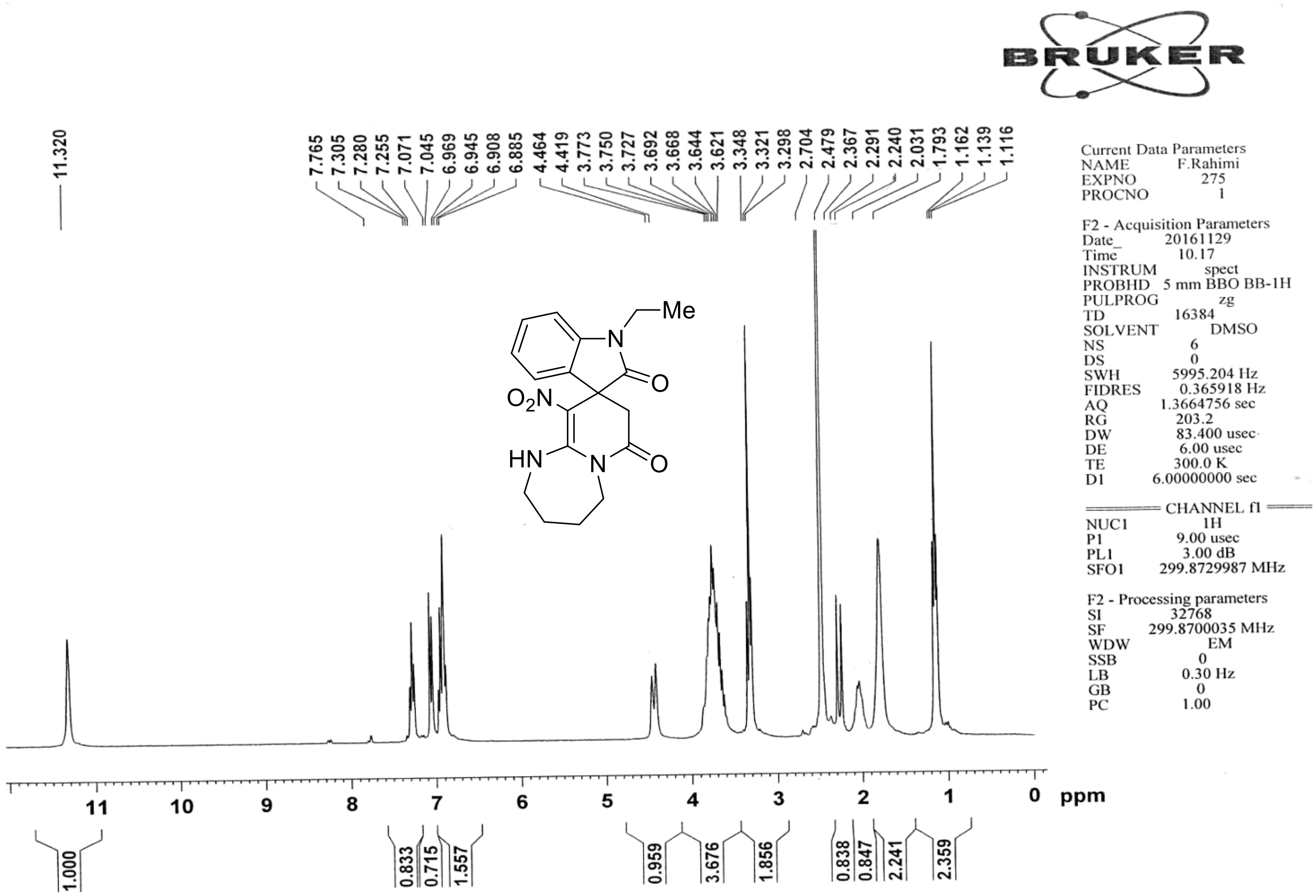

BRUKER
11.320
7.765 7.305 7.280 7.255 7.071 7.045 6.969 6.945 6.908 6.885 4.464 4.419 3.773 3.750 3.727 3.692 3.668 3.644 3.621 3.348 3.321 3.298 2.704 2.479 2.367 2.291 2.240 2.031 1.793 1.162 1.139 1.116
Current Data Parameters
NAME F.Rahimi
EXPNO 275
PROCNO 1
F2 - Acquisition Parameters
Date_ 20161129
Time 10.17
INSTRUM spect
PROBHD 5 mm BBO BB-1H
PULPROG zg
TD 16384
SOLVENT DMSO
NS 6
DS 0
SWH 5995.204 Hz
FIDRES 0.365918 Hz
AQ 1.3664756 sec
RG 203.2
DW 83.400 usec
DE 6.00 usec
TE 300.0 K
D1 6.00000000 sec
CHANNEL f1
NUC1 1H
P1 9.00 usec
PL1 3.00 dB
SFO1 299.8729987 MHz
F2 - Processing parameters
SI 32768
SF 299.8700035 MHz
WDW EM
SSB 0
LB 0.30 Hz
GB 0
PC 1.00
Me
N
O
O2N
HN
N
O
11 10 9 8 7 6 5 4 3 2 1 0 ppm
1.000 0.833 0.715 1.557 0.959 3.676 1.856 0.838 0.847 2.241 2.359

^{1}H NMR of 5g

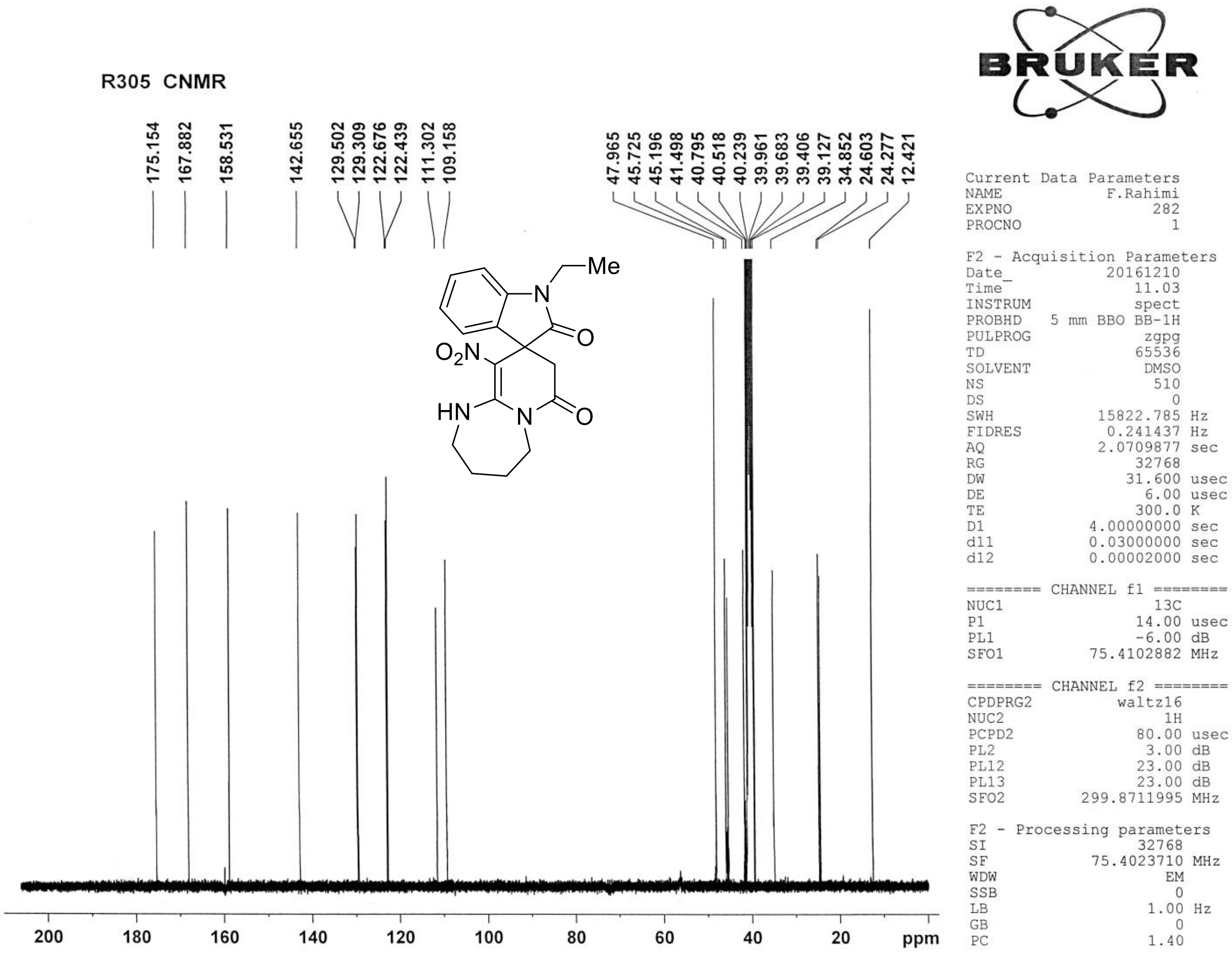
R305 CNMR
175.154
167.882
158.531
142.655
129.502
129.309
122.676
122.439
111.302
109.158
47.965
45.725
45.196
41.498
40.795
40.518
40.239
39.961
39.683
39.406
39.127
34.852
24.603
24.277
12.421
Me
N
O
O$_2$N
HN
N
O
BRUKER
Current Data Parameters
NAME F.Rahimi
EXPNO 282
PROCNO 1
F2 - Acquisition Parameters
Date_ 20161210
Time 11.03
INSTRUM spect
PROBHD 5 mm BBO BB-1H
PULPROG zgpg
TD 65536
SOLVENT DMSO
NS 510
DS 0
SWH 15822.785 Hz
FIDRES 0.241437 Hz
AQ 2.0709877 sec
RG 32768
DW 31.600 usec
DE 6.00 usec
TE 300.0 K
D1 4.00000000 sec
d11 0.03000000 sec
d12 0.00002000 sec
======== CHANNEL f1 ========
NUC1 13C
P1 14.00 usec
PL1 -6.00 dB
SFO1 75.4102882 MHz
======== CHANNEL f2 ========
CPDPRG2 waltz16
NUC2 1H
PCPD2 80.00 usec
PL2 3.00 dB
PL12 23.00 dB
PL13 23.00 dB
SFO2 299.8711995 MHz
F2 - Processing parameters
SI 32768
SF 75.4023710 MHz
WDW EM
SSB 0
LB 1.00 Hz
GB 0
PC 1.40
200
180
160
140
120
100
80
60
40
20
ppm

^{13}C NMR of 5g

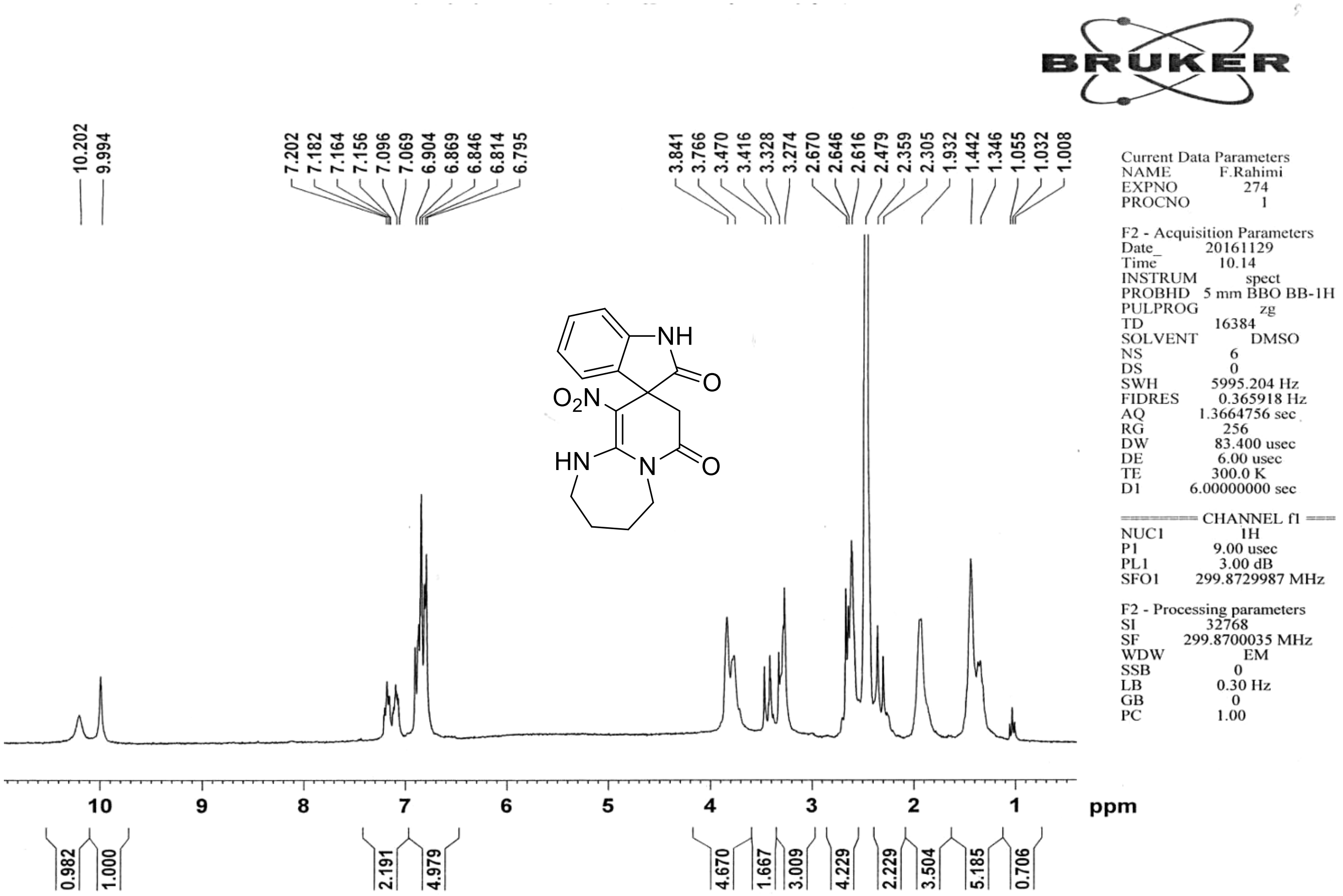
BRUKER
10.202
9.994
7.202
7.182
7.164
7.156
7.096
7.069
6.904
6.869
6.846
6.814
6.795
3.841
3.766
3.470
3.416
3.328
3.274
2.670
2.646
2.616
2.479
2.359
2.305
1.932
1.442
1.346
1.055
1.032
1.008
NH
O
O_2N
HN
N
O
Current Data Parameters
NAME F.Rahimi
EXPNO 274
PROCNO 1
F2 - Acquisition Parameters
Date_ 20161129
Time 10.14
INSTRUM spect
PROBHD 5 mm BBO BB-1H
PULPROG zg
TD 16384
SOLVENT DMSO
NS 6
DS 0
SWH 5995.204 Hz
FIDRES 0.365918 Hz
AQ 1.3664756 sec
RG 256
DW 83.400 usec
DE 6.00 usec
TE 300.0 K
D1 6.00000000 sec
CHANNEL f1
NUC1 1H
P1 9.00 usec
PL1 3.00 dB
SFO1 299.8729987 MHz
F2 - Processing parameters
SI 32768
SF 299.8700035 MHz
WDW EM
SSB 0
LB 0.30 Hz
GB 0
PC 1.00
10
9
8
7
6
5
4
3
2
1
ppm
0.982
1.000
2.191
4.979
4.670
1.667
3.009
4.229
2.229
3.504
5.185
0.706

^{1}H NMR of 5h

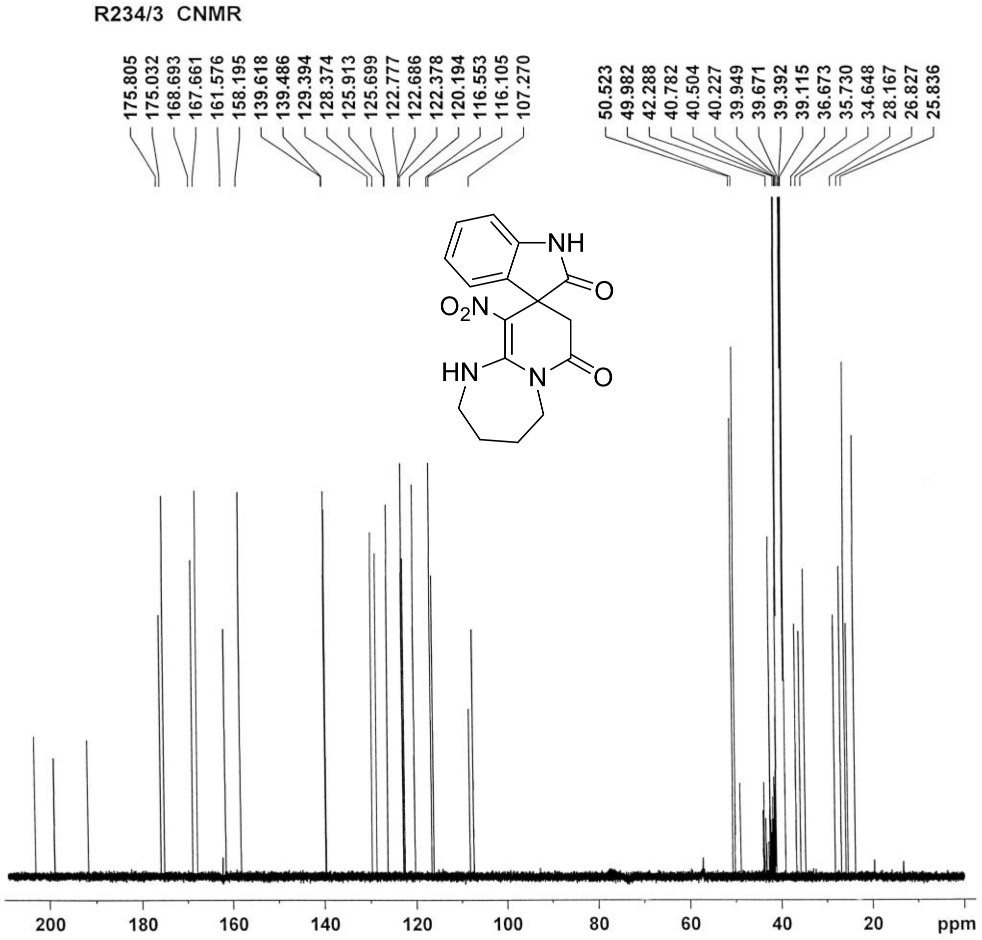


```
Current Data Parameters
NAME            F.Rahimi
EXPNO                281
PROCNO                 1

F2 - Acquisition Parameters
Date_           20161210
Time               10.03
INSTRUM            spect
PROBHD   5 mm BBO BB-1H
PULPROG             zgpg
TD                 65536
SOLVENT             DMSO
NS                  1200
DS                     0
SWH           15822.785 Hz
FIDRES         0.241437 Hz
AQ            2.0709877 sec
RG                 32768
DW               31.600 usec
DE                 6.00 usec
TE                300.0 K
D1           4.00000000 sec
d11          0.03000000 sec
d12          0.00002000 sec

======== CHANNEL f1 ========
NUC1                 13C
P1                 14.00 usec
PL1                -6.00 dB
SFO1          75.4102882 MHz

======== CHANNEL f2 ========
CPDPRG2          waltz16
NUC2                  1H
PCPD2              80.00 usec
PL2                 3.00 dB
PL12               23.00 dB
PL13               23.00 dB
SFO2         299.8711995 MHz

F2 - Processing parameters
SI                 32768
SF            75.4023710 MHz
WDW                   EM
SSB                    0
LB                  1.00 Hz
GB                     0
PC                  1.40
```

^{13}C NMR of 5h

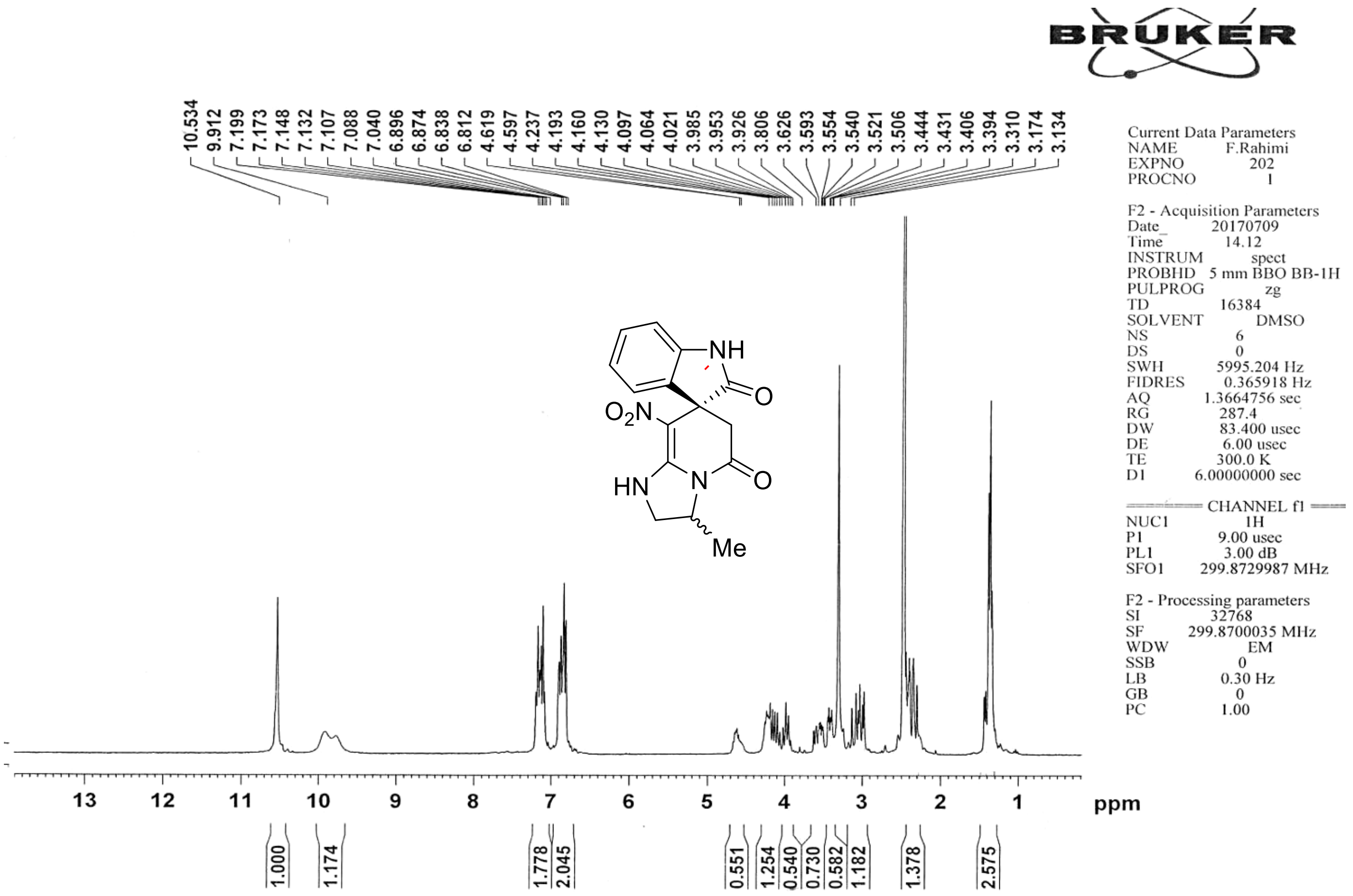
BRUKER
10.534
9.912
7.199
7.173
7.148
7.132
7.107
7.088
7.040
6.896
6.874
6.838
6.812
4.619
4.597
4.237
4.193
4.160
4.130
4.097
4.064
4.021
3.985
3.953
3.926
3.806
3.626
3.593
3.554
3.540
3.521
3.506
3.444
3.431
3.406
3.394
3.310
3.174
3.134
NH
O
O2N
HN
N
O
Me
Current Data Parameters
NAME F.Rahimi
EXPNO 202
PROCNO 1
F2 - Acquisition Parameters
Date_ 20170709
Time 14.12
INSTRUM spect
PROBHD 5 mm BBO BB-1H
PULPROG zg
TD 16384
SOLVENT DMSO
NS 6
DS 0
SWH 5995.204 Hz
FIDRES 0.365918 Hz
AQ 1.3664756 sec
RG 287.4
DW 83.400 usec
DE 6.00 usec
TE 300.0 K
D1 6.00000000 sec
CHANNEL f1
NUC1 1H
P1 9.00 usec
PL1 3.00 dB
SFO1 299.8729987 MHz
F2 - Processing parameters
SI 32768
SF 299.8700035 MHz
WDW EM
SSB 0
LB 0.30 Hz
GB 0
PC 1.00
13 12 11 10 9 8 7 6 5 4 3 2 1 ppm
1.000
1.174
1.778
2.045
0.551
1.254
0.540
0.730
0.582
1.182
1.378
2.575

^{1}H NMR of 5i

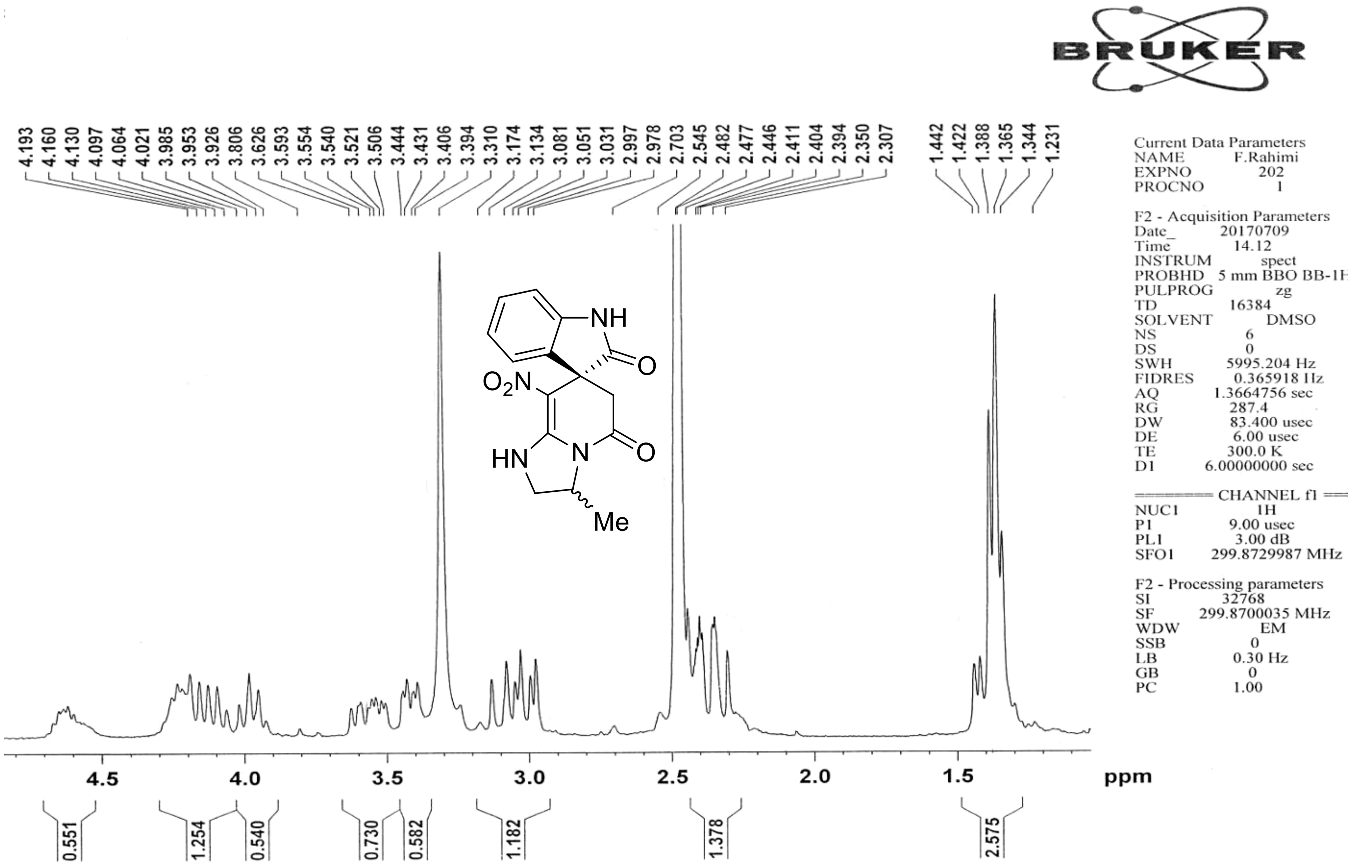

^{1}H expand of 5i

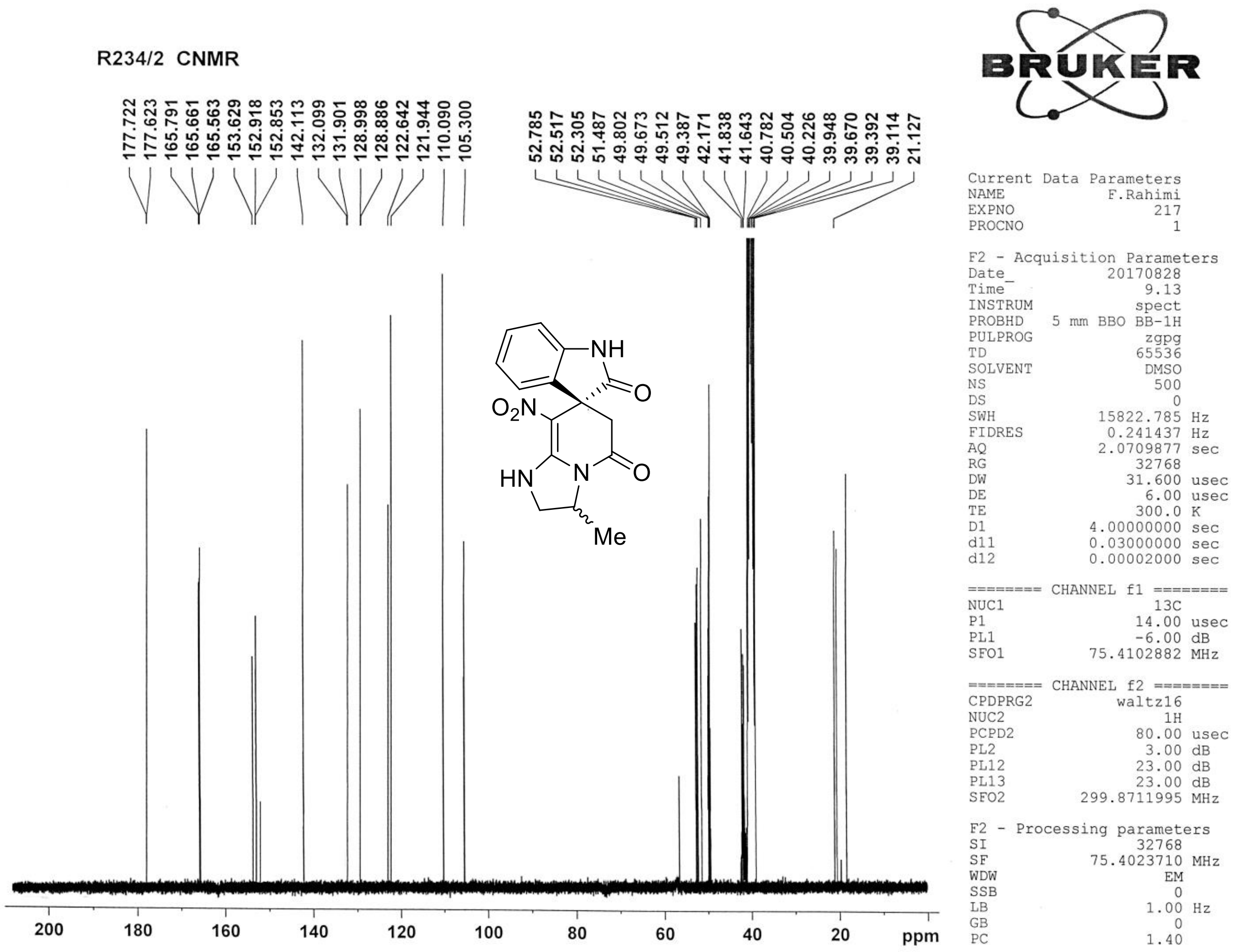

R234/2 CNMR
177.722
177.623
165.791
165.661
165.563
153.629
152.918
152.853
142.113
132.099
131.901
128.998
128.886
122.642
121.944
110.090
105.300
52.785
52.517
52.305
51.487
49.802
49.673
49.512
49.387
42.171
41.838
41.643
40.782
40.504
40.226
39.948
39.670
39.392
39.114
21.127
NH
O
O2N
HN
N
O
Me
200
180
160
140
120
100
80
60
40
20
ppm
BRUKER
Current Data Parameters
NAME F.Rahimi
EXPNO 217
PROCNO 1
F2 - Acquisition Parameters
Date_ 20170828
Time 9.13
INSTRUM spect
PROBHD 5 mm BBO BB-1H
PULPROG zgpg
TD 65536
SOLVENT DMSO
NS 500
DS 0
SWH 15822.785 Hz
FIDRES 0.241437 Hz
AQ 2.0709877 sec
RG 32768
DW 31.600 usec
DE 6.00 usec
TE 300.0 K
D1 4.00000000 sec
d11 0.03000000 sec
d12 0.00002000 sec
======== CHANNEL f1 ========
NUC1 13C
P1 14.00 usec
PL1 -6.00 dB
SFO1 75.4102882 MHz
======== CHANNEL f2 ========
CPDPRG2 waltz16
NUC2 1H
PCPD2 80.00 usec
PL2 3.00 dB
PL12 23.00 dB
PL13 23.00 dB
SFO2 299.8711995 MHz
F2 - Processing parameters
SI 32768
SF 75.4023710 MHz
WDW EM
SSB 0
LB 1.00 Hz
GB 0
PC 1.40

^{13}C NMR of 5i

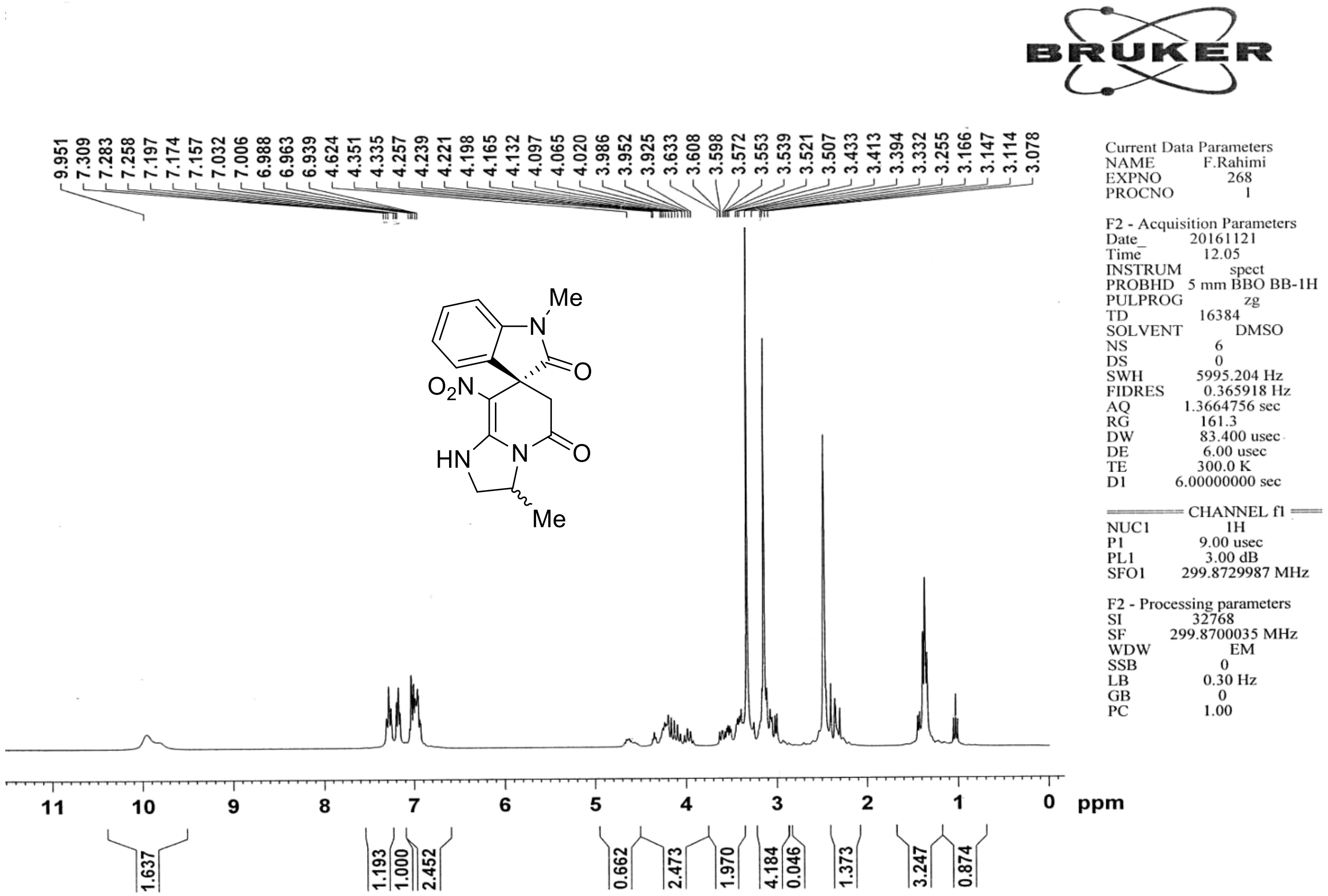
BRUKER
9.951
7.309
7.283
7.258
7.197
7.174
7.157
7.032
7.006
6.988
6.963
6.939
4.624
4.351
4.335
4.257
4.239
4.221
4.198
4.165
4.132
4.097
4.065
4.020
3.986
3.952
3.925
3.633
3.608
3.598
3.572
3.553
3.539
3.521
3.507
3.433
3.413
3.394
3.332
3.255
3.166
3.147
3.114
3.078
Me
N
O
O_2N
HN
N
O
Me
Current Data Parameters
NAME F.Rahimi
EXPNO 268
PROCNO 1
F2 - Acquisition Parameters
Date_ 20161121
Time 12.05
INSTRUM spect
PROBHD 5 mm BBO BB-1H
PULPROG zg
TD 16384
SOLVENT DMSO
NS 6
DS 0
SWH 5995.204 Hz
FIDRES 0.365918 Hz
AQ 1.3664756 sec
RG 161.3
DW 83.400 usec
DE 6.00 usec
TE 300.0 K
D1 6.00000000 sec
CHANNEL f1
NUC1 1H
P1 9.00 usec
PL1 3.00 dB
SFO1 299.8729987 MHz
F2 - Processing parameters
SI 32768
SF 299.8700035 MHz
WDW EM
SSB 0
LB 0.30 Hz
GB 0
PC 1.00
11 10 9 8 7 6 5 4 3 2 1 0 ppm
1.637
1.193
1.000
2.452
0.662
2.473
1.970
4.184
0.046
1.373
3.247
0.874

^{1}H NMR of 5j

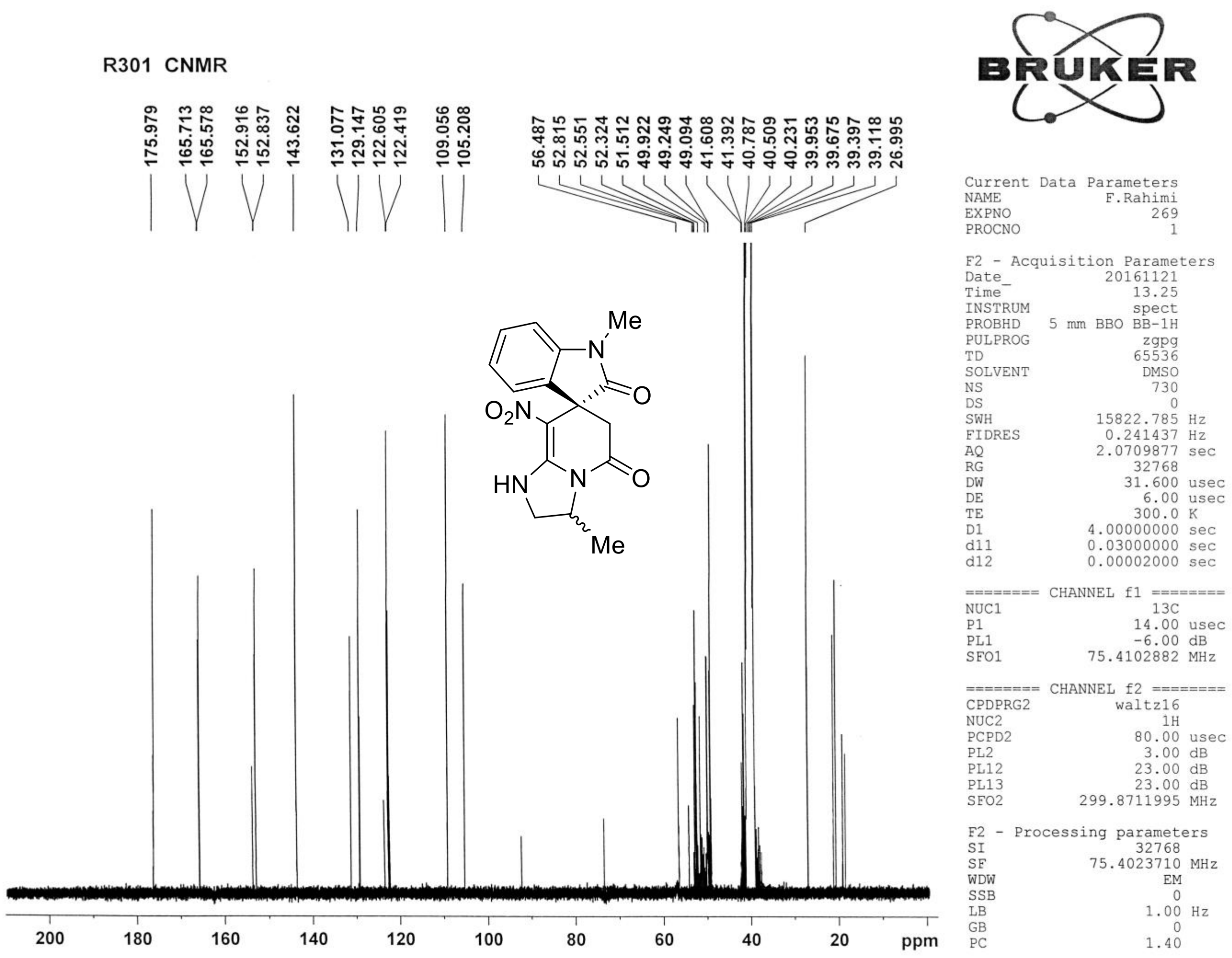
R301 CNMR
175.979
165.713
165.578
152.916
152.837
143.622
131.077
129.147
122.605
122.419
109.056
105.208
56.487
52.815
52.551
52.324
51.512
49.922
49.249
49.094
41.608
41.392
40.787
40.509
40.231
39.953
39.675
39.397
39.118
26.995
BRUKER
Me
N
O
O_2N
HN
N
O
Me
200
180
160
140
120
100
80
60
40
20
ppm
Current Data Parameters
NAME F.Rahimi
EXPNO 269
PROCNO 1
F2 - Acquisition Parameters
Date_ 20161121
Time 13.25
INSTRUM spect
PROBHD 5 mm BBO BB-1H
PULPROG zgpg
TD 65536
SOLVENT DMSO
NS 730
DS 0
SWH 15822.785 Hz
FIDRES 0.241437 Hz
AQ 2.0709877 sec
RG 32768
DW 31.600 usec
DE 6.00 usec
TE 300.0 K
D1 4.00000000 sec
d11 0.03000000 sec
d12 0.00002000 sec
======== CHANNEL f1 ========
NUC1 13C
P1 14.00 usec
PL1 -6.00 dB
SFO1 75.4102882 MHz
======== CHANNEL f2 ========
CPDPRG2 waltz16
NUC2 1H
PCPD2 80.00 usec
PL2 3.00 dB
PL12 23.00 dB
PL13 23.00 dB
SFO2 299.8711995 MHz
F2 - Processing parameters
SI 32768
SF 75.4023710 MHz
WDW EM
SSB 0
LB 1.00 Hz
GB 0
PC 1.40

^{13}C NMR of 5j
